# miR-21-5p/PRKCE axis implicated in immune infiltration and poor prognosis of kidney renal clear cell carcinoma

**DOI:** 10.3389/fgene.2022.978840

**Published:** 2022-09-13

**Authors:** Jinxiang Wang, Jie Jin, Yanling Liang, Yihe Zhang, Nisha Wu, Mingming Fan, Fangyin Zeng, Fan Deng

**Affiliations:** ^1^ Department of Cell Biology, School of Basic Medical Sciences, Southern Medical University, Guangzhou, China; ^2^ Department of Clinical Laboratory, the Fifth Affiliated Hospital, Southern Medical University, Guangzhou, China; ^3^ Department of Clinical Laboratory, Nanfang Hospital, Southern Medical University, Guangzhou, China

**Keywords:** kidney renal clear cell carcinoma, prognostic biomarker, nomogram, hypermethylation, immune infiltration

## Abstract

Kidney renal clear cell carcinoma (KIRC or ccRCC) is the most notorious subtype of renal cell carcinoma for its poor prognosis. Mounting evidence has highlighted the key role of PRKCE in the initiation and development of several types of human cancer, including kidney renal clear cell carcinoma (KIRC). However, the mechanism of PRKCE aberrant expression and the specific clinical correlation of PRKCE expression with immune cell infiltration in KIRC remains elusive. Therefore, we analyzed the relationship between PRKCE and KIRC using many databases, including Oncomine, TCGA, GTEx, TIMER, and GEO. We found that PRKCE decreased in KIRC tumor tissue compared to normal tissue. The Kaplan-Meier Plotter analysis and Univariate and Multivariate Cox analyses were used to evaluate the association between PRKCE and clinicopathological variables and prognosis. Low PRKCE expression was associated with poor survival and histologic grade, T stage, pathologic stage, and M stage. Besides, the C-indexes and calibration plots of the nomogram based on multivariate analysis showed an effective predictive performance for KIRC patients. In addition, PRKCE may be positively correlated with inflammation and negatively correlated with proliferation, metastasis, and invasion as identified by CancerSEA. Moreover, overexpression of PRKCE suppressed ACHN and Caki-1 cell proliferation, migration, and invasion *in vitro*. Additionally, methylation level data acquired from UALCAN, DiseaseMeth, CCLE, LinkedOmics, and MEXPRESS was used to investigate the relationship between PRKCE expression and PRKCE methylation level. Furthermore, upstream potential miRNA predictions were further performed to explore the mechanism of PRKCE decreased expression in KIRC using multiple online databases available on publicly assessable bioinformatics platforms. High PRKCE methylation levels and hsa-miR-21-5p may contribute to PRKCE low expression in KIRC. Finally, an analysis of immune infiltration indicated that PRKCE was associated with immune cell infiltration. Importantly, PRKCE may affect prognosis partially by regulating immune infiltration in KIRC. In summary, PRKCE may serve as a novel prognostic biomarker reflecting immune infiltration level and a novel therapeutic target in KIRC.

## Introduction

Renal cell carcinoma (RCC) is a prevalent cancer worldwide, with about 5 and 3% new incidence rates in males and females, respectively (Siegel et al., 2021). The most typical subtype of RCC is Kidney Renal Clear Cell Carcinoma (KIRC), which represents nearly 75% of kidney cancers (Rini et al., 2009). Advanced KIRC patients have a poor prognosis due to intrinsic resistance to radiotherapy and chemotherapy (Makhov et al., 2018). Moreover, distant metastasis occurs in numerous patients at diagnosis, limiting the surgical treatment of KIRC (Gupta et al., 2008). In addition, despite early surgical treatment, up to 30% of patients eventually relapse and develop metastases (Hutson and Figlin, 2007; Hsieh et al., 2017). All these cause a poor survival rate in advanced patients, especially when the 5-year survival rate is only 10–20% (Hsieh et al., 2018; Li et al., 2018). Thus, exploring novel therapeutic targets and prognostic markers for KIRC is urgent.

Protein kinase C (PKC) is a serine/threonine kinase that regulates an adverse set of cellular processes, including proliferation, apoptosis, cell survival, and migration. There is substantial evidence linking PKC to tumorigenesis (Griner and Kazanietz, 2007). PRKCE encodes the protein kinase C epsilon (PKC-ε), regulating various physiological functions (Gorin and Pan, 2009; Toton et al., 2011). PKC-ε, an oncogenic member of the novel PKC family, is aberrantly expressed and associated with poor clinical outcomes in numerous epithelial tumors (Basu, 2020). For renal cell carcinoma, PKC-ε expression and activation contribute to cell invasion and stem cell pathogenesis in renal cell carcinoma (Engers et al., 2000; Huang et al., 2016). The prognostic value and the potential mechanism of PRKCE aberrant expression in KIRC remain not fully investigated, although PKC epsilon (ε) was upregulated in KIRC and was associated with tumor Fuhrman grade and T stage in clear cell RCCs (Huang et al., 2011).

The tumor microenvironment (TME), including stromal cells, immune cells, chemokines, and cytokines (Zhang et al., 2020), plays a vital role in immunotherapies, especially immune checkpoint blockade (Li et al., 2021). Immunotherapy is an emerging strategy for patients with multiple cancers, including melanoma (Exley et al., 2017) and renal cell carcinoma (Li Y. et al., 2020). For the KIRC, only a subset of patients benefited from low response rates (Sharpe and Pauken, 2018; Li Y. et al., 2020). Accumulating evidence has demonstrated that some immune cells, such as CD8^+^T cells, regulatory T cells, and mast cells, play a vital role in KIRC progression (Fu et al., 2017; Siska et al., 2017; Chen et al., 2019). KIRC has the distinctive characteristics of an immunogenic tumor, including circulating tumor-specific T cells and cytotoxic T cells, which confirm and selectively kill tumor cells (Nukui et al., 2017). Some studies reveal that PRKCE is associated with macrophage and dendritic cell (DC) activation and regulates T-cell migration (Aksoy et al., 2004; Ong et al., 2014). Meanwhile, PKC epsilon (ε) was a critical component of the TLR-4 signaling pathway and played a key role in macrophages and dendritic cells (DC) (Aksoy et al., 2004). Nevertheless, the association of PRKCE with KIRC-infiltrating immune cells needs further investigation.

In this study, we used multiple bioinformatics and statistical approaches to evaluate the correlation of PRKCE expression with prognosis in KIRC patients with various clinical-pathological characteristics, together with the potential functions of PRKCE in KIRC. A nomogram was further construed to predict the patient’s prognosis. The PRKCE function in KIRC was analyzed by CancerSEA. We further overexpressed PRKCE *in vitro* to detect the effect on the ability of proliferation, invasion, and migration of KIRC cells. Methylation analysis and upstream potential miRNA predicts were further performed to explore the mechanism of PRKCE aberrant low expression in KIRC. Moreover, we investigated the effect of PRKCE on immune infiltration in KIRC. Our study found the important role of PRKCE in KIRC and provided an underlying mechanism of PRKCE decreased expression and a potential relationship with tumor-immune infiltrations.

## Materials and methods

### Oncomine database analysis

The expression level of the *prkce* gene in various types of cancers was identified in the Oncomine database (Rhodes et al., 2007) (https://www.oncomine.org/resource/login.html). The threshold was determined according to the following values: *p*-value of 0.001, fold change of 1.5, and gene ranking in the top 10%.

### Data preprocessing

RNA expression data and clinical information from KIRC patients (including 539 tumors and 72 normal tissues) were obtained from TCGA. Four mRNA microarray datasets (GSE71963, GSE46699, GSE53757, and GSE36895) were downloaded from the GEO database. GSE71963 covered 32 KIRC tissue samples and 16 adjacent non-tumor samples. GSE46699 contained 67 KIRC tissue samples and 63 adjacent non-tumor samples. GSE53757 included 72 pairs of KIRC tissue samples and adjacent non-tumor samples. GSE36895 contained 29 KIRC tissue samples and 23 adjacent non-tumor samples. The log2 [TPM+1] transformed PRKCE expression data of 539 KIRC patients was further analyzed. The mutation status of PRKCE was analyzed by the cBioPortal for Cancer Genomics (http://www.cbioportal.org/). The TISIDB database was employed to explore the expression of PRKCE and clinical features across various cancers (Ru et al., 2019). The copy number variation (CNV) data of PRKCE were obtained from the cBioPortal online platform (https://www.cbioportal.org/).

### TIMER database analysis

TIMER is a web tool for in-depth integrative analysis of tumor-immune interactions in various cancer types (https://cistrome.shinyapps.io/timer/) (Li et al., 2017). PRKCE expression in various types of cancer was analyzed by using TIMER2.0 (http://timer.comp-genomics.org/) (Li T. et al., 2020). The “Diff Exp” module was applied to assess PRKCE gene expression differences between tumor and normal tissues. The “Gene module” was employed in our research to investigate the correlation between PRKCE and immune cell infiltration in KIRC. *p* < 0.05 was considered statistically significant.

### The GEDS database analysis

GEDS is an online server that displays human gene expression in cancer types, normal tissues, and cell lines. We used the GEDS database (http://bioinfo.life.hust.edu.cn/web/GEDS/) (Xia et al., 2019) to explore the expression level of PRKCE in the tumor cell lines of 29 tissues.

### UALCAN analysis

UALCAN (http://ualcan.path.uab.edu/analysis-prot.html) (Chandrashekar et al., 2017) is an online tool that provides comprehensive analyses of transcriptome data from The Cancer Genome Atlas (TCGA). In the “TCGA Gene analysis” segment, we used the gene symbol(s) “PRKCE” segment, selected “kidney renal clear cell carcinoma” and methylation segment to explore the PRKCE methylation level. We also used a CPTAC segment to assess protein levels of PRKCE between normal tissues and the KIRC in UALCAN.

### Single-cell analysis

CancerSEA (http://biocc.hrbmu.edu.cn/CancerSEA/home.jsp), a web server for comprehensive analysis of functional states of cancer cells at a single-cell resolution (Yuan et al., 2019), was applied to explore the functional heterogeneity of PRKCE in KIRC cells.

### Cell culture

The ACHN and Caki-1 human RCC cell lines were bought from the American Type Culture Collection, maintained in the suggested media, and incubated at 37°C in a 5% CO2 incubator.

### Overexpression of PRKCE

The plasmid vector and PRKCE plasmid were constructed by GenePharma (GenePharma, Suzhou, China), and cell transfection has been described previously (Xu et al., 2018). Plasmids were transfected into cells using Hilymax (Dojindo, H357), following the manufacturer’s instructions.

### Quantitative real-time polymerase chain reaction

Total RNAs were isolated using Trizol reagent (Invitrogen). One μg of total RNA was subjected to reverse transcription using HiScript II Q RT SuperMix for qPCR (Vazyme, R222-01, China). Quantitative Real-time PCR (qRT-PCR) was conducted using a Step One Plus TM Real-Time PCR system with ChamQ/SYBR qPCR Master Mix (Vazyme, Q311-03, China) to determine the mRNA expression level of PRKCE. Expression levels were normalized to the GAPDH level. The PCR primers used were as follows:

*GAPDH* forward: 5′**-**GTC​TCC​TCT​GAC​TTC​AAC​AGC​G**-**3′ and Reverse: 5′-ACC​ACC​CTG​TTG​CTG​TAG​CCA​A**-**3′; *PRKCE* Forward 5′-AGC​CTC​GTT​CAC​GGT​TCT​ATG​C**-**3′ and Reverse: 5′**-**GCA​GTG​ACC​TTC​TGC​ATC​CAG​A**-**3′.

### Cell proliferation assay

Cell counting kit-8 (CCK8) was used to assay the proliferative abilities. For cell proliferation experiments, 1,000 cells were seeded into each well of 96-well plates with three replicates for ACHN and Caki-1. Absorbance at 450 nm was detected using the cell counting kit-8 assay (Dojindo, CK04, Japan) at 1, 3, 5, and 7 days post-seeding.

### Wound healing assay

When confluence reached 80–90% in 6-well plates, a 10 μl pipette tip was used to scratch the monolayer. Then, KIRC cells were washed and starved to migrate for the indicated time. Images were taken at 0, 24, and 36 h using the microscope and analyzed by ImageJ.

### Cell invasion and migration assay

Cell invasion assays were conducted using 8 µm pore size chambers coated with matrigel gel (Corning, 354480, United States). 1.5 × 10^4^ cells were resuspended in serum-free medium and seeded into the upper chambers; the bottom chambers were filled with medium containing serum. After incubation for 24 h, the invasive cells were stained and images were captured using the microscope. Three random fields were analyzed for each chamber. Cell migration assays were performed using chambers without matrigel gel (Corning, 3,422, United States). 1 × 10^4^ cells were resuspended in serum-free medium, and other procedures were the same as above.

### Functional enrichment and analysis of immune cell infiltration

The online GeneMANIA database (http://www.genemania.org) (Warde-Farley et al., 2010) was employed to construct the PRKCE interaction network. The STRING online database (https://string-db.org/) (Szklarczyk et al., 2011; Szklarczyk et al., 2021) was applied to construct a protein-protein interaction (PPI) network of PRKCE. We used a protein name (“PRKCE”) and organism (“*Homo sapiens*”) to search the STRING database. The following key parameters were set: the minimum required interaction score [the mean of network edges (“evidence”)], “medium confidence (0.400),” and the maximum number of interactors to display (“no more than 10 interactors” in the first shell). Gene ontology (GO) enrichment and Kyoto Encyclopedia of Genes and Genomes (KEGG) pathway analyses of co-expression genes were demonstrated by the “ClusterProfiler” R package (Yu et al., 2012) and visualized by the “ggplot2” R package (Ginestet, 2011). The relative tumor infiltration levels of 24 immune cell types were quantified by ssGSEA to interrogate expression levels of genes in published signature gene lists (Bindea et al., 2013). The signatures we presented here included various sets of innate and adaptive immune cell types and 509 genes in all. The Wilcoxon rank-sum test and Spearman correlation were used to explore the correlation between PRKCE expression and the infiltration levels of immune cells.

### StarBase database analysis

StarBase (http://starbase.sysu.edu.cn/) (Li et al., 2014) is an online tool for investigating upstream miRNAs. The expression level of hsa-miR-21-5p in KIRC and normal controls was also analyzed by starBase. In the “Pan-Cancer” module, we used the “miRNA differential expression” segment and inputted “has-miR-21-5p ”, selected “KIRC” to search for the expression level of has-miR-21-5p between cancer and normal.

### The miRNA prediction

Several target gene prediction databases, including TargetScan, microT, PITA, RNA22, miRmap, PicTar, and miRanda, were applied to predict candidate binding miRNAs. Only the predicted miRNAs that commonly appeared in more than two databases, as mentioned above, were included for subsequent analyses. Besides, miRactDB was used to predict the relationship between candidate binding miRNAs and PRKCE. (https://ccsm.uth.edu/miRactDB/index.html) (Tan H. et al., 2021). TargetScan (https://www.targetscan.org/vert_80/) was used to predict the binding site between candidate miRNA and PRKCE.

### Methylation analysis of PRKCE

The UALCAN (http://ualcan.path.uab.edu/analysis-prot.html) and the human disease methylation database DiseaseMeth version 2.0 database (http://bio-bigdata.hrbmu.edu.cn/diseasemeth/) were utilized to assess methylation levels of PRKCE between the KIRC and normal tissues. CCLE (https://portals.broadinstitute.org/ccle) (Vasaikar et al., 2018; Ghandi et al., 2019), LinkedOmics (https://linkedomics.or), and MEXPRESS (https://mexpress.be) (Koch et al., 2015; Koch et al., 2019) were used to explore the association between PRKCE gene expression and its methylation. In the MEXPRESS database, we used the “enter a gene name” segment and inputted “*PRKCE*”, selecting “KIRC” to investigate the methylation level in KIRC.

### Kaplan-Meier Plotter analysis

Kaplan-Meier plotter (http://kmplot.com/analysis/) (Lanczky and Gyorffy, 2021), an online database capable of accessing the effects of genes or miRNAs on survival in different cancers, including KIRC, was performed for prognosis analyses based on the expression levels of PRKCE in KIRC in related immune cell subgroup. Log-rank *p* < 0.05 was considered statistically significant.

### Statistical analysis

The statistical analysis in this study was automatically calculated by the online tool mentioned above. Results were expressed as the mean ± SD of three independent experiments unless otherwise specified. The data were analyzed by Student’s t-test between any two groups. *p* < 0.05 or log-rank *p* < 0.05 was considered as statistically significant.

## Results

### Correlation of PRKCE expression with cancer risk factors and aggressiveness in KIRC patients

**FIGURE 1:**
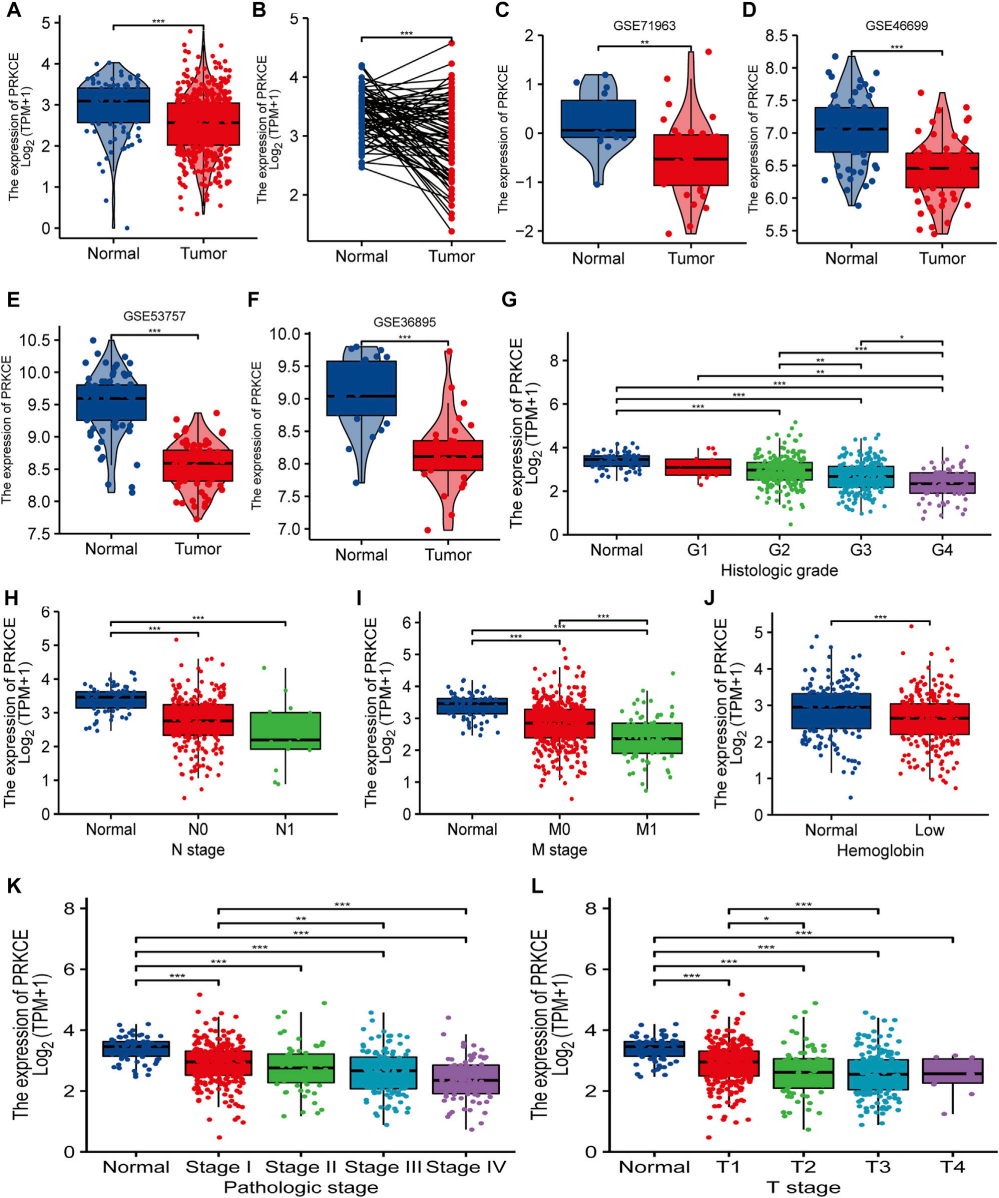
Decreased PRKCE expression correlates with cancer risk factors and aggressiveness in KIRC patients. **(A–F)** The expression levels of PRKCE in KIRC. **(G–L)** Association between PRKCE expression and clinicopathological characteristics including **(G)** histologic grade, **(H)** N stage, **(I)** M stage, **(J)** Hemoglobin level, **(K)** Pathologic stage, **(L)** T stage. *p < 0.05, **p < 0.01, ***p < 0.001. KIRC, Kidney Renal Clear Cell Carcinoma.

PRKCE expression in KIRC samples and normal tissues was analyzed using TCGA and GTEx. The results showed that expression levels of PRKCE in 531 tumor tissues were lower than in 100 normal samples (*p* < 0.001; Figure 1A). We also applied the Oncomine database and TIMER to analyze the expression levels of PRKCE across different cancer types. The Oncomine database indicated (Supplementary Figure S1A) that PRKCE mRNA levels were significantly lower in most human cancers, including kidney cancer. Supplementary Figure S1B highlights the same result as above, derived from data derived from TIMER. Furthermore, a markedly lower PRKCE expression in KIRC was observed in 72 paired tumor samples compared with 72 adjacent normal samples (*p* < 0.001; Figure 1B).

Additionally, PRKCE was generally lower in many tumor cell lines, including kidney tumor cell lines (Supplementary Figure S1C). Consistently, from four KIRC studies of the GEO database, PRKCE was expressed lowly in KIRC compared to non-cancer tissues (Figures 1C–F). We also analyzed the correlation between PRKCE expression and the pathological stages of tumors by using the TISIDB database. TISIDB database analysis demonstrated that decreased expression of PRKCE was most correlated with tumor grade and stage (Supplementary Figure S1D,E) in KIRC. In addition, to confirm the significant role of PRKCE expression in KIRC, RNA sequence data from 539 KIRC patients (186 female and 353 male) with completed patient characteristics were downloaded from the TCGA database. We sought to analyze the association between PRKCE expression and clinical-pathological in 539 KIRC. As shown in Figure 1G–L and Supplementary Table S1, decreased expression of PRKCE was observed in histologic grade 4, N1 stage, M1 stage, low hemoglobin, pathologic stage IV, and T4 stage compared to that of normal controls. Moreover, univariate logistic regression was employed to evaluate the association between the expression of PRKCE and prognostic factors. Decreased expression of PRKCE was related to T stage (OR = 0.413 for T3 vs. T1), M stage (OR = 0.375 for m1 vs. m0), Pathologic stage (OR = 0.524 for stage II & stage III vs. stage I), histologic grade (OR = 0.261 for G4 vs. G1), and gender (OR = 0.436 for male vs. female) significantly (all *p* < 0.05) (Supplementary Table S2). These findings show that PRKCE expression is downregulated in kidney renal clear cell carcinoma and suggest that decreased expression of PRKCE may play a vital regulatory role in kidney renal clear cell carcinoma progression.

### Decreased expression of PRKCE is associated with poor prognosis in KIRC

**FIGURE 2:**
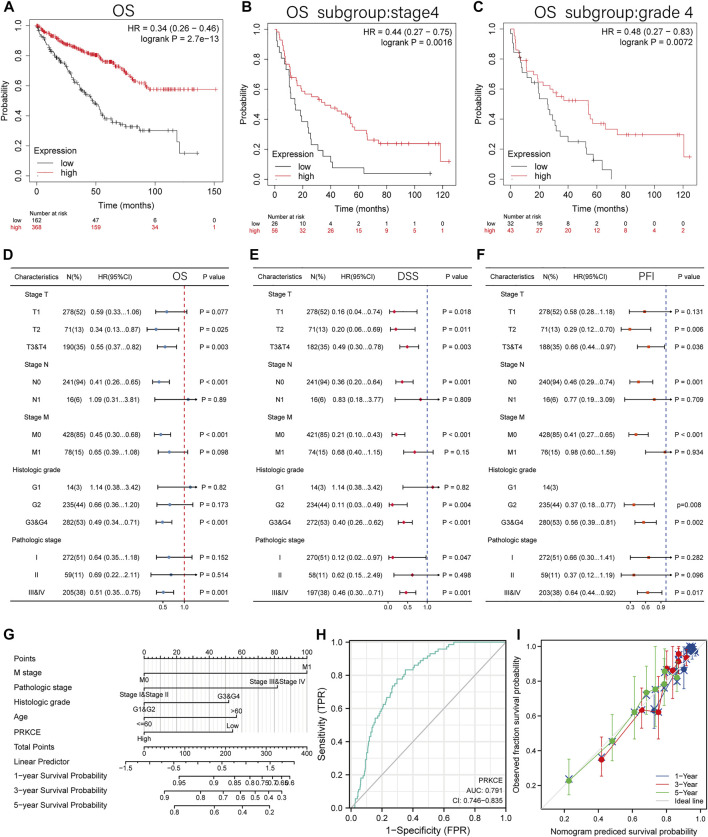
The prognosis value of the PRKCE expression in KIRC. **(A)** Kaplan-Meier overall survival (OS) curves comparing the low and high expression of PRKCE in KIRC in Kaplan-Meier Plotter. **(B–C)** OS Survival curves of stage4 and grade4 subgroups comparing the low and high expression of PRKCE in KIRC in Kaplan-Meier Plotter. **(D–F)** Forest plots displaying clinical subgroup analyses of KIRC prognosis (OS, DSS, and PFI) in the TCGA database. **(G)** A nomogram that integrates PRKCE and other prognosis factors in KIRC from TCGA data. **(H)** Receiver operating characteristic (ROC) curve for PRKCE expression in KIRC. **(I)** Calibration plots of the nomogram for estimating the probability of OS at 1, 3, and 5 years

To better understand the prognostic value and potential mechanism of PRKCE expression in KIRC, an overall survival analysis for PRKCE in various cancers was conducted by TISIDB. As shown in Supplementary Figure S2A, decreased expression of PRKCE was most correlated with poor overall survival (OS) (*p* = 1.06e-08) in KIRC. We used cox proportional hazards models to investigate the effect of PRKCE on cancer prognosis in 33 cancer types. The correlations of cox regression analysis between PRKCE and overall survival (OS), disease-specific survival (DSS), and progression-free interval (PFI) were shown in forest charts. As shown in Supplementary Figure S2B–D, decreased PRKCE expression was most related to a poor OS, DSS, PFI in KIRC [OS: *n* = 539, HR = 0.453, 95% CI = 0.330–0.620, *p* < 0.001; DSS (disease-specific survival): *n* = 528, HR = 0.283, 95% CI = 0.182–0.440, *p* < 0.001; PFI (progression-free interval):*n* = 537, HR = 0.404, 95% CI = 0.289–0.564, *p* < 0.001]. Similarly, we also identified that low PRKCE expression was related to poor overall survival (OS: HR = 0.34, 95% CI from 0.26 to 0.46, log-rank *p* = 2.7e-13) (Figure 2A) in the Kaplan-Meier plotter. We further displayed subgroup analyses of prognosis in the Kaplan-Meier plotter, which suggested that survival rates of KIRC patients with low PRKCE expression were poor in the stage 4 (OS: HR = 0.44, 95% CI from 0.27 to 0.75, log-rank *p* = 0.0016) and grade 4 (OS: HR = 0.48 95% CI from 0.27 to 0.83, log-rank *p* = 0.0072) subgroup of overall survival (Figure 2B,C).

Univariate cox regression analysis also demonstrated that decreased expression of PRKCE was related to short OS (HR: 0.453; CI: 0.330–0.660; *p* < 0.001) (Supplementary Table S3). Furthermore, to better investigate the characteristics related to prognosis, an analysis of multivariate regression was performed with the N stage, T stage, M stage, histologic grade, and pathologic stage. Similarly, lower expression of PRKCE was still an independent factor related to poor OS (HR: 0.571; CI: 0.357–0.914; *p* = 0.020) (Supplementary Table S3). Based on the results of multivariate regression, we analyzed the effect of PRKCE expression on prognosis (DSS, PFI, and OS) in various subgroups. We found that lower expression of PRKCE was poor OS in T2 (HR: 0.34; CI: 0.13–0.87; *p* = 0.025), N0 subgroup (HR: 0.41; CI: 0.26–0.65; *p* < 0.001), M0 subgroup (HR: 0.45; CI: 0.30–0.68; *p* < 0.001) (Figure 2D). The DSS and PFI subgroup analysis consistently showed that KIRC patients with low expression of PRKCE had a shorter survival in a subgroup of other factors (Figure 2E,F). These results indicate that decreased expression of PRKCE is intimately related to an unfavorable prognosis in KIRC.

To provide a quantitative approach to predicting the prognosis of KIRC patients, we performed a nomogram based on PRKCE and other independent clinical risk factors. As shown in Figure 2G, for instance, a KIRC patient with low PRKCE risk (54 points), M1 (100 points), stage III (82 points), G2 (0 points), and ≤60 (0 points) received a total point score of 236. The survival rates of 1-, 3-, 5-years were about 83.5, 60, and 41%. The ROC curve was performed to evaluate the diagnostic value of PRKCE in KIRC. As displayed in (Figure 2H), the ROC curve analysis indicated that PRKCE had a certain accuracy (AUC = 0.791, CI = 0.746–0.835) in predicting KIRC. At a cutoff of 3.030, PRKCE had an accuracy, sensitivity, and specificity of 67.1, 83.3, and 64.9. We further evaluated the efficiency of the nomogram, and the results indicated that the C-index of the model was 0.760 (CI: 0.742–0.778) (Figure 2I), which suggested that the prediction efficiency of this model is moderately accurate and PRKCE could be a promising biomarker to discriminate kidney renal clear carcinoma from adjacent controls.

### The function of the PRKCE in KIRC

**FIGURE 3:**
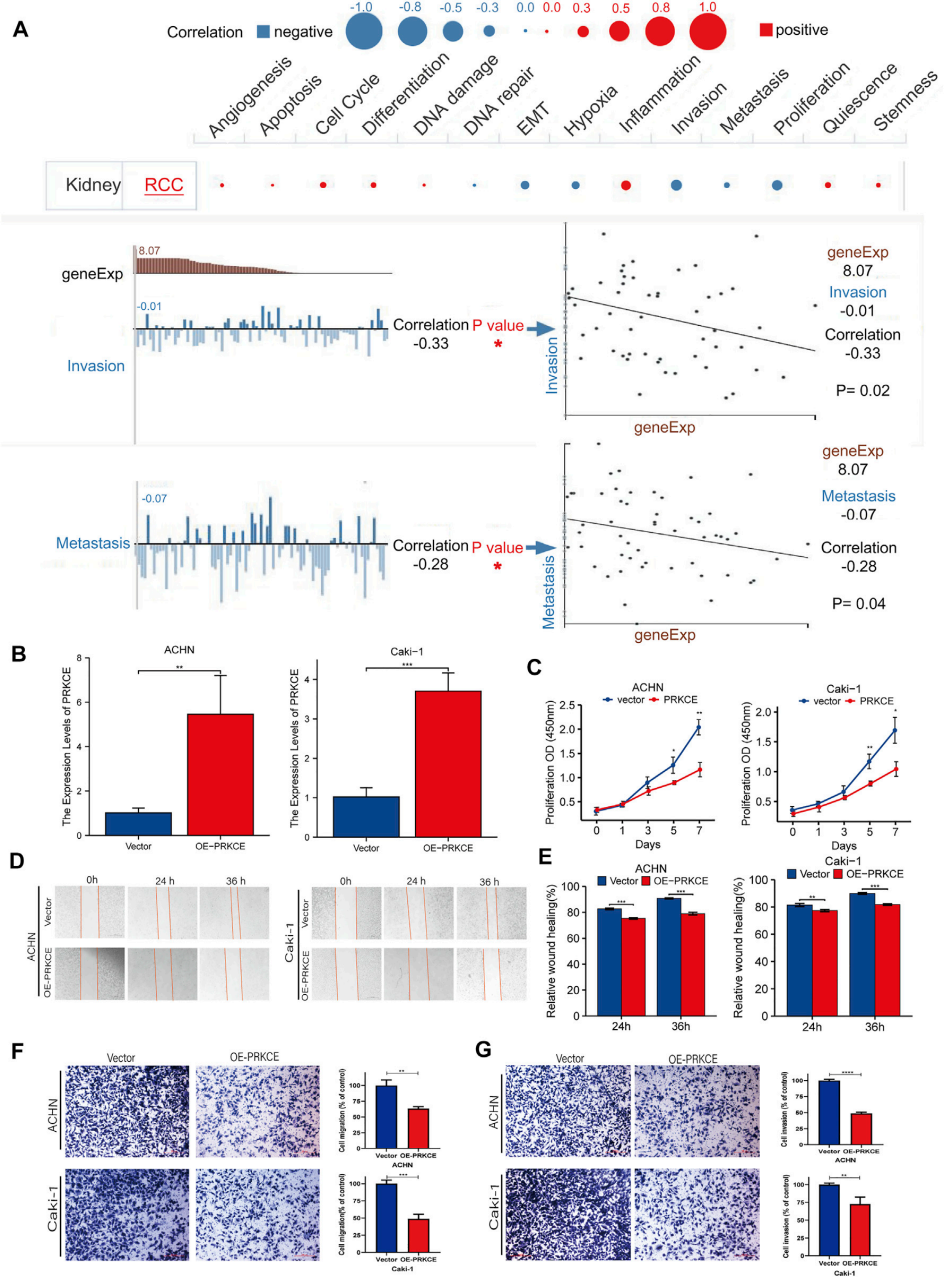
PRKCE overexpression suppresses proliferation, migration, and invasion of kidney renal clear cell carcinoma cells *in vitro*. **(A)** Single-cell analysis showed the functional state of PRKCE in KIRC. CancerSEA analysis demonstrated that PRKCE negatively correlated with regulating proliferation, migration, and invasion. **(B)** The mRNA level of PRKCE in kidney renal clear cell carcinoma cells was detected by qRT-PCR. **(C)** The CCK8 assays showed that PRKCE overexpression attenuated the proliferation of kidney renal clear cell carcinoma cells. **(D,E)** Wound healing assays demonstrated that PRKCE overexpression inhibited the migration ability of kidney renal clear cell carcinoma cells. **(F, G**) The migration and invasion abilities of ACHN and Caki-1 were weakened after overexpressing PRKCE.

To investigate the role of PRKCE in KIRC, we performed a single-cell analysis using CancerSEA. The results showed that PRKCE positively correlated with inflammation and negatively correlated with proliferation, invasion (*p =* 0.02), and metastasis (*p =* 0.04) in KIRC cells (Figure 3A). To further validate the results of the single-cell analysis, we explored the role of PRKCE overexpression in the two clear-cell renal cell carcinoma cell lines. Efficiency was detected using qRT-PCR (Figure 3B). The proliferative curves of CCK8 assays indicated that cell proliferation was inhibited by PRKCE overexpression in ACHN and Caki-1 cell lines (Figure 3C). The wound-healing assay and cell migration experiment indicated that PRKCE overexpression suppressed the migration ability of KIRC cells (Figure 3D–F). Similarly, the cell invasion assay showed that PRKCE overexpression suppressed the invasion ability of KIRC cells (Figure 3G).

To better explore the functions of PRKCE in cancer, we constructed the gene-gene interaction network for PRKCE and the altered neighboring genes using Gene MANIA. The results indicated that the 20 most frequently altered genes were closely correlated with PRKCE, including RHOC, EGFR, TICAM2, PRKD1, and SEC14L5 (Supplementary Figure S3A). Furthermore, a protein-protein interaction (PPI) network of PRKCE was generated using the STRING database. There were 39 edges and 11 nodes, including PDPK1, IKBKG, IKBKB, and TIAM1, with a PPI enrichment *p* value = 0.000115 (Supplementary Figure S3B). The correlation heatmap between the two cohorts is shown in (Supplementary Figure S3C). The correlation analyses between the expression of PRKCE and co-expression genes in kidney renal clear cell carcinoma (KIRC) from TCGA were shown in (Supplementary Figure S3D–K). In addition, the functional analysis indicated that these genes were significantly associated with the inflammatory and immune responses, such as the chemokine signaling pathway, T cell receptor signaling pathway, and Fc receptor signaling pathway (Supplementary Figure S3L). To better analyze the biological enrichment process of PRKCE-associated genes, we used the “Cluster Profiler” R package and the “ggplot2″ R package for GO and KEGG pathway analyses, which demonstrated that GO enrichment in the biological process of PRKCE was associated with the Fc receptor signaling pathway (*p* = 1.10e-13), immune response-activating cell surface-receptor signaling pathway (*p* = 2.10e-13), innate immune response activating cell-surface receptor signaling pathway (*p* = 3.81e-09), activation of innate immune response and (*p* = 1.02e-08) and immune response-regulating cell surface-receptor signaling pathway involved in phagocytosis (*p* = 9.48e-09). KEGG pathway functional annotations indicated that PRKCE was involved in the chemokine signaling pathway (*p* = 1.37e-11), T cell receptor signaling pathway (*p* = 1.52e-11), and PD-L1 expression and PD-1 checkpoint pathway in cancer (*p* = 6.37e-08) (Supplementary Table S4). These results strongly imply that PRKCE regulates the immune response in kidney renal clear cell carcinoma cancer.

### Correlation between expression of PRKCE and methylation

**FIGURE 4:**
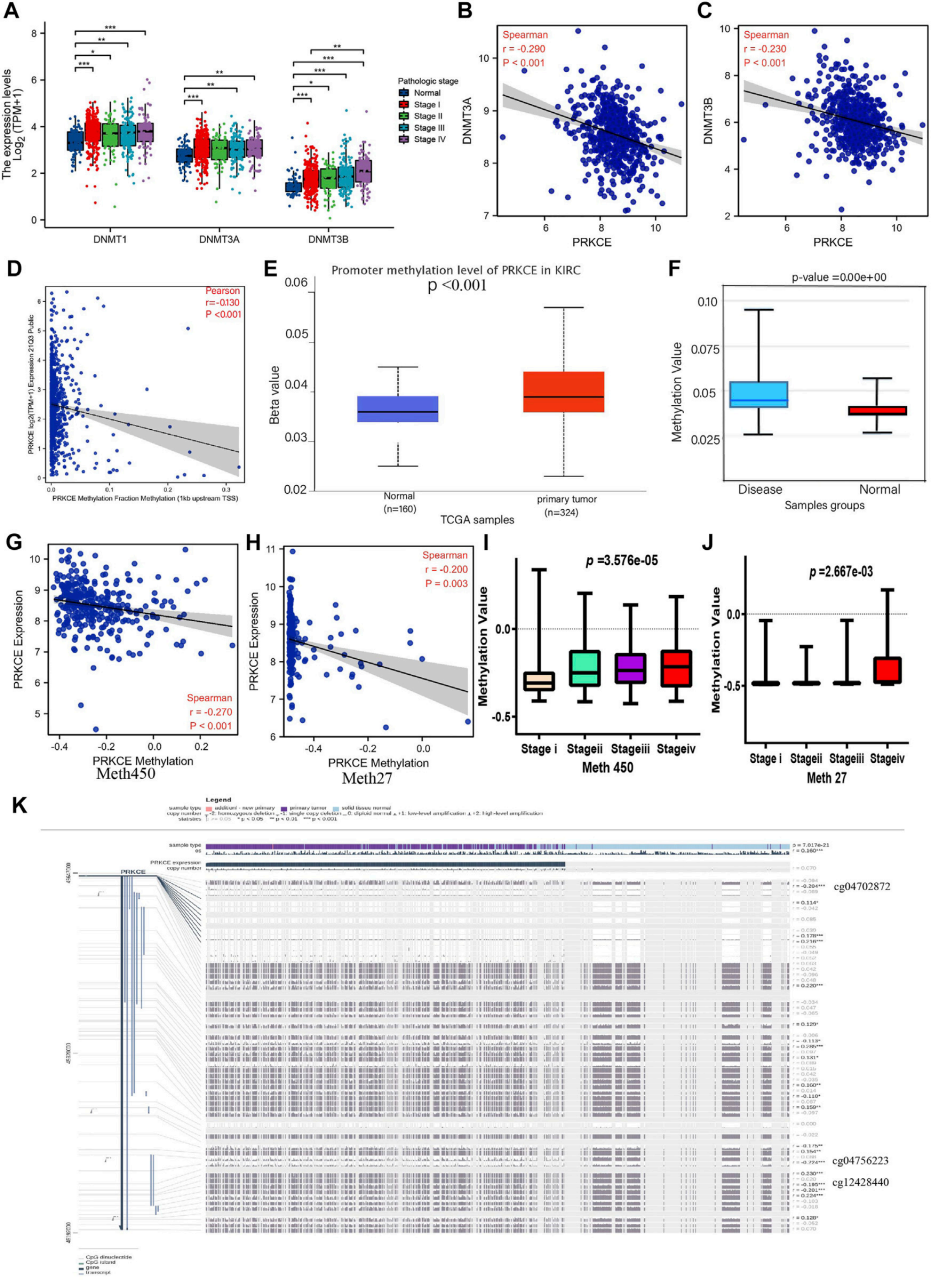
Methylation analysis of PRKCE in KIRC. **(A)** DNMT1, DNMT3A, DNMT3B expression in different pathological stages of KIRC. **(B,C)** The correlation between PRKCE expression and DNMT3A and DNMT3B was analyzed using the LinkedOmics database. **(D)** The correlation between PRKCE methylation and expression level analysis using CCLE data in kidney cancer cells. **(E)** The promoter methylation level of PRKCE in tumor tissues (*n* = 324) and normal tissues (*n* = 160) from UALCAN data. **(F)** Methylation level was evaluated using the Disease Meth version 2.0 database. **(G,H)** The correlation between PRKCE methylation and the PRKCE expression level was analyzed using Illumina 450 and 27K data in KIRC. **(I,J)** Methylation was evaluated using IIIumina 450 and 27K data in different pathological stages. **(K)** The methylation site of PRKCE DNA sequence association with gene expression was visualized using MEXPRESS.

To investigate the potential abnormal downregulated mechanism of PRKCE in KIRC, we first applied cBioPortal to validate the relationship between mRNA expression of PRKCE and its copy number variation (CNV) in KIRC. As shown in Supplementary Figure S4A, no significant association was observed between PRKCE expression and copy number variation in KIRC, suggesting that CNV may not affect the decreased expression of PRKCE. Hence, we further explored the correlation of mRNA expression of PRKCE with *PRKCE* methylation in KIRC. As shown in Figure 4A and Supplementary Figure S4B, the mRNA levels of DNA methyltransferases (including DNMT1, DNMT3A, and DNMT3B) were significantly higher in KIRC patients and correlated with tumor stage and histologic tumor grade than in normal. Meanwhile, the LinkedOmics database analysis showed that the mRNA expression of PRKCE was significantly negatively related to the expression of DNMT3A (R = -0.29, *p* < 0.001) and DNMT3B (R = -0.23, *p* < 0.001) (Figures 4B,C). Moreover, the CCLE dataset analysis also showed that PRKCE methylation negatively correlated with PRKCE expression in various cancer cells, including kidney cancer cells (R = -0.13, *p* < 0.001) (Figure 4D and Supplementary Figure S4C). Meanwhile, the analysis of the UALCAN database (*p* < 0.001; Figure 4E) indicated that the promoter methylation level of PRKCE was significantly higher in KIRC samples than in the normal tissues from the TCGA database. Similarly, Diseasemeth version 2.0 analysis indicated that the methylation of PRKCE was significantly higher in KIRC compared with normal tissues (Figure 4F). LinkedOmics integrated analysis demonstrated a significantly inversely relationship of PRKCE DNA methylation in Illumina 450K array (Figure 4G) and 27K array (Figure 4H) with PRKCE transcriptional expression and were significantly related to tumor stage (Figures 4I,J) in KIRC patients. In addition, we found three methylation sites (cg04702872, cg04756223, and cg12428440) in the DNA sequences of PRKCE that were negatively associated with their expression levels (Figure 4K). The methylation levels of the PRKCE heatmap were shown in KIRC by MethSurv analysis (Supplementary Figure S4D). These data indicate that decreased PRKCE expression might be related to PRKCE’s high methylation level.

### Prediction and analysis of upstream miRNAs of PRKCE

**FIGURE 5:**
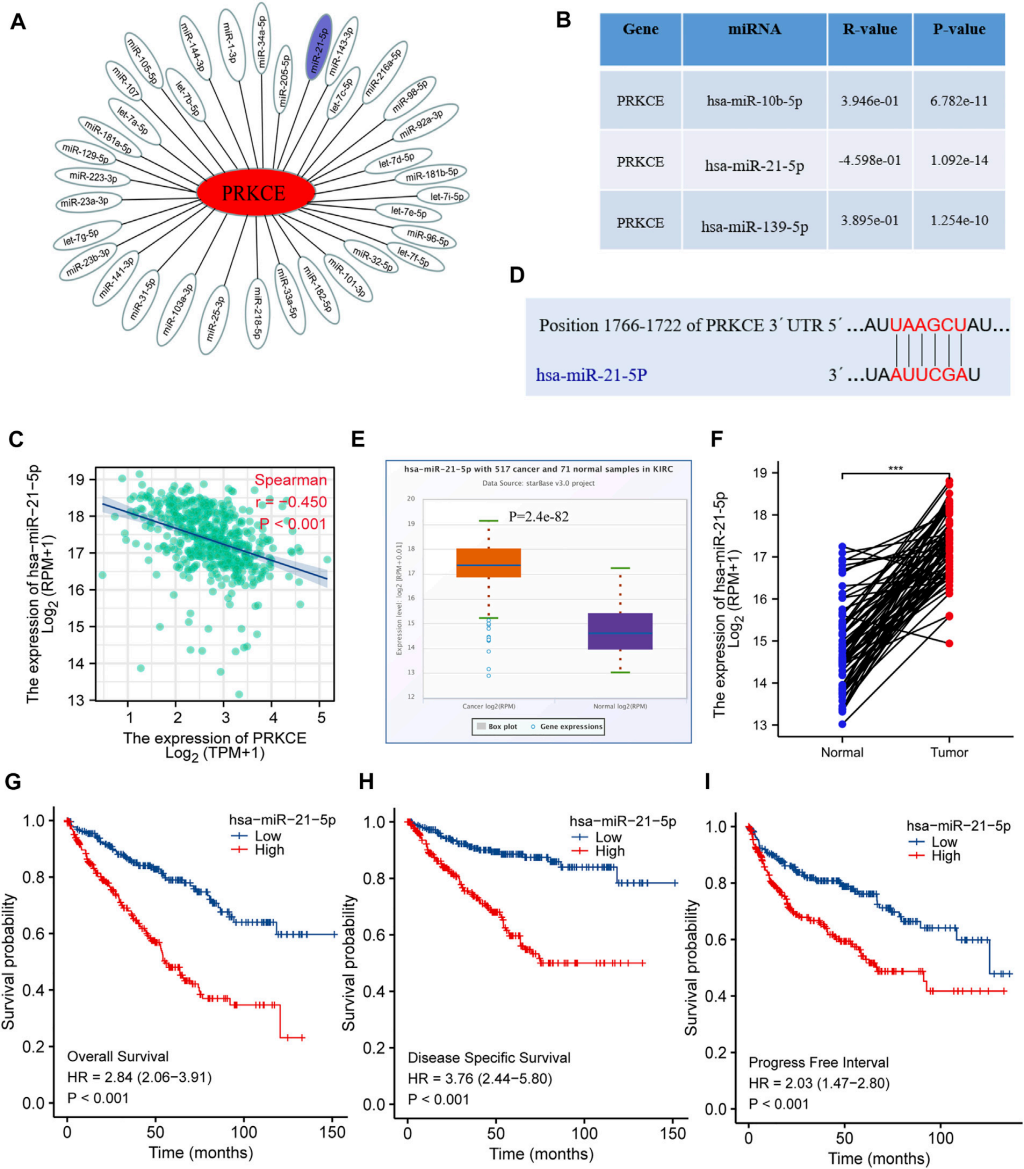
Identification of hsa-miR-21-5p as a potential upstream miRNA of PRKCE in KIRC. **(A)** The miRNA-PRKCE regulatory network. **(B)** The predicted miRNAs of PRKCE were analyzed by miRactDB. **(C)** Scatter plots and correlation analysis between hsa-miR-21-5p and PRKCE in KIRC. **(D)** The target bind site in the PRKCE 3′UTR was predicted by TargetScan. **(E)** The expression of hsa-miR-21-5p in KIRC and control normal samples was investigated by the starBase database. **(F)** TCGA database and statistical analyses of hsa-miR-21-5p expression in 71 pairs of KIRC tissues and adjacent normal tissues. **(G–I)** Survival curves of OS, DSS, and PFI between low and high expression of hsa-miR-21-5p in KIRC.

To further confirm whether some potential ncRNAs regulated PRKCE low expression, the starBase database was applied to predict upstream miRNAs that may bind to PRKCE. As shown in Figure 5A, 35 miRNAs can bind to mRNA upstream of PRKCE, including miR-21-5p. Meanwhile, miRactDB analysis showed that mRNA of PRKCE correlated with 3 miRNAs, including miR-21-5p, miR-216a-5p, and miR-92a-3p. Furthermore, miRactDB analysis showed that mRNA of PRKCE was significantly positively correlated with miR-10b-5p (R = 0.39, *p* = 6.782e-11) and miR-139-5p (R = 0.39, *p* = 1.254e-10) in KIRC (Figure 5B). Conversely, PRKCE was significantly negatively correlated with miR-21-5p (R = -0.45, *p* < 0.001) (Figure 5C), also, the bind sites analysis between PRKCE and miR-21-5p was predicted by TargetScan (Figure 5D). Compared with normal tissues, high hsa-miR-21-5p expressions were observed in the KIRC tumor (Figure 5E). High expression of hsa-miR-21-5p was also confirmed in a pairwise comparison of 71 KIRC tissues with matched adjacent benign tissues derived from the TCGA miRNAseq (Figure 5F). We further analyzed the relationship between hsa-miR-21-5p expression and the survival of KIRC patients. Higher hsa-miR-21-5p expression indicated a worse prognosis in KIRC patients (Figures 5G–I). These results suggest that hsa-miR-21-5p might be the most potential regulatory miRNA of PRKCE in KIRC.

### Effects of PRKCE on immune cell infiltration

**FIGURE 6:**
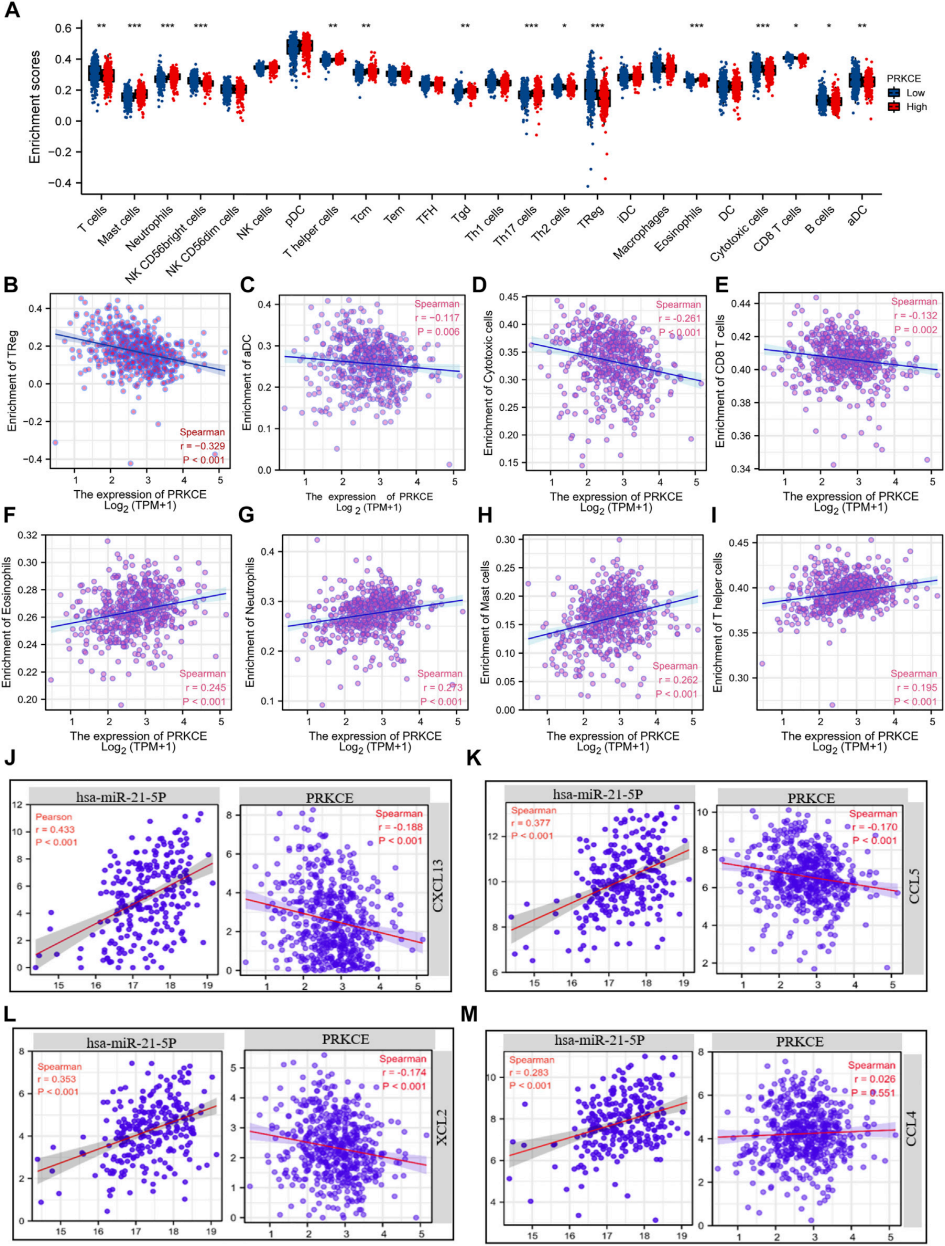
Chemokines mediated the regulatory effects of PRKCE on immune cell infiltration. **(A)** Proportions of the 24 tumor-infiltrating immune cell types in low and high expression groups of PRKCE. **(B–E)** PRKCE expression significantly negatively correlates with infiltrating levels of **(B)** Treg, **(C)** aDC, **(D)** Cytotoxic, and **(E)** CD8 T cells. **(F–I)** PRKCE expression significantly positively correlates with infiltrating levels of **(F)** Eosinophils, **(G)** Neutrophils, **(H)** Mast cells, and **(I)** T helper cells. **(J–M)** Pearson’s correlation analysis was applied to explore the relationships between the hsa-miRNA-21-5p/PRKCE axis and 4 Chemokines (CXCL13, CCL5, XCL2, and CCL4).

Since decreased expression of PRKCE plays a vital role in regulating the immune response in KIRC (Supplementary Figure S3L). To fully investigate the potential relationship between PRKCE expression and immune infiltrations in KIRC. We conducted the “SCNA” module to explore the correlation between somatic CNA and the abundance of immune infiltrates by using TIMER2.0. As shown in Supplementary Figure S5A, infiltration levels of immune cells, including B cells, CD8^+^T cells, CD4^+^ T cells, macrophages, neutrophils, and dendritic cells (DCs), seemed to be associated with altered PRKCE gene copy numbers. The Spearman correlation analysis quantified by ssGSEA has been applied to demonstrate the association between the immune cell infiltration level and expression level (TPM) of PRKCE to assess the effect of PRKCE on the tumor microenvironment (TME). Kidney renal clear cell carcinoma samples were divided into PRKCE high- and low-expression groups. Among the 24 subpopulations of immune cells, among the 15 immune cells affected by PRKCE expression, mast cells (*p* < 0.001), neutrophils (*p* < 0.001), T helper cells (*p* = 0.004), and eosinophils (*p* < 0.001), Th17 cells (*p* < 0.001), and Tcm (*p* = 0.004) levels were apparently decreased in the PRKCE low-expression group compared with high-expression group (Figure 6A). Conversely, Treg (*p* < 0.001) was increased in the PRKCE low-expression group compared with the high-expression group (Figure 6A). Interestingly, the expression of PRKCE was negatively associated with tumor-infiltration immune effector cells from both innate and adaptive immune systems, including Treg cells (R = -0.330, *p* < 0.001), aDC (R = -0.117, *p* = 0.006), cytotoxic cells (R = -0.261, *p* < 0.001), CD8 T cells (R = -0.132, *p* = 0.002) (Figures 6B–E) and positively associated with eosinophils, neutrophils, mast cells, and T helper cells (Figures 6F–I). Moreover, PRKCE expression was significantly negatively correlated with the infiltration of macrophages, CD8^+^ T cells, Tregs, and MDSC, but not with tumor purity by TIMER2.0 analysis (Supplementary Figure S5B–E). Meanwhile, the ImmuneScore and EstimateScore in KIRC also showed that the low PRKCE group exhibited higher levels of the ImmuneScore and EstimateScore than the high PRKCE group in KIRC, and Spearman correlation analysis indicated that the expression of PRKCE was negatively correlated with ImmuneScore (R = −0.220, *p* < 0.001) and EstimateScore (R = -0.100, *p* = 0.019) (Supplementary Figure S5F,G).

Chemokine changes, including CCL4, CCL5, CXCL13, and XCL2, have been shown to have high expression and correlate with immune infiltration cells in KIRC (Tan P. et al., 2021). Given that PRKCE contributes to activating the chemokine signaling pathway (Figure 4D), we sought to investigate whether hsa-miR-21-5p and PRKCE are related to the release of the above chemokines. Co-expression analysis of hsa-miR-21-5p-chemokines and PRKCE-chemokines demonstrated that hsa-miR-21-5p was positively related to CCL4, CCL5, CXCL13, and XCL2 (Figure 6J–M). On the contrary, the expression of PRKCE was negatively correlated with CCL5, CXCL13, and XCL2 (all *p* < 0.05) (Figure 6J–L). These results strongly imply that low expression of PRKCE might be involved in reduced anti-cancer immune infiltration and even less responsive to immunotherapy in the kidney renal clear cell carcinoma microenvironment.

### Correlation of the decreased expression of PRKCE with various checkpoints in KIRC

**FIGURE 7:**
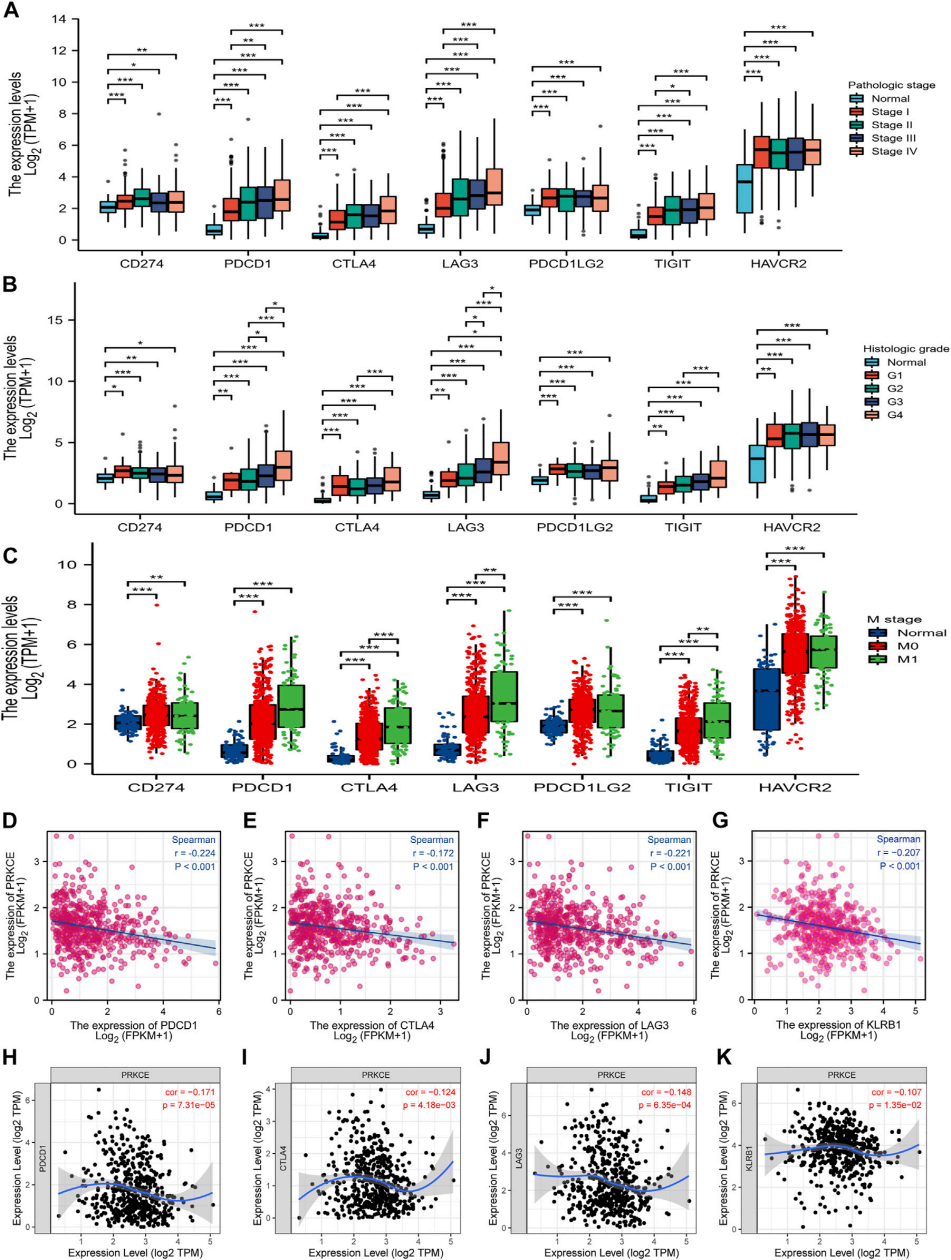
The relationship between PRKCE expression and immune checkpoint genes in KIRC. The expression of CD274 (PDL1), PDCD1 (PD1), CTLA-4, LAG3, PDCD1LG2 (PDL2), TIGIT, and HAVCR2 (TIM-3) in **(A)** pathologic stage I, II, III; **(B)** histologic grade G1, 2, 3, 4 and **(C)** M stage M0, M1. **(D–G)** PRKCE expression was negatively correlated with the checkpoint molecules in KIRC. Markers include PDCD1 (PD1), CTLA4, LAG3, and KLRB1. **(H–K)** The expression correlation of PRKCE with PDCD1, CTLA4, LAG3, and KLRB1 in KIRC was investigated by TIMER.

Given that PRKCE may regulate PD-L1 expression by KEGG enrichment analysis, we next investigated the expression level of immune T cell checkpoints in the KIRC patients and normal groups by the TCGA database. As shown in Figures 7A–C, the expression of CD274 (PDL1), PDCD1(PD1), CTLA-4, LAG3, PDCD1LG2 (PDL2), TIGIT, and HAVCR2 (TIM-3) in pathologic stage I, II, III; histologic grade G1, 2, 3, 4 and M stage M0, M1 KIRC group were markedly increased compared with that in the normal group. Spearman correlation analysis demonstrated that PRKCE expression was significantly negatively correlated with PDCD1, CTLA-4, LAG3, and KLRB1 in KIRC (Figures 7D–G). Similar results were obtained by TIMER dataset analysis (Figures 7H–K). These findings further demonstrate that PRKCE expression is significantly correlated with immune cell infiltration and suggest that PRKCE plays a vital role in tumor-immune evasion in the kidney renal clear cell carcinoma cancer microenvironment.

### Prognostic analysis of PRKCE expression based on immune cells in KIRC

**FIGURE 8:**
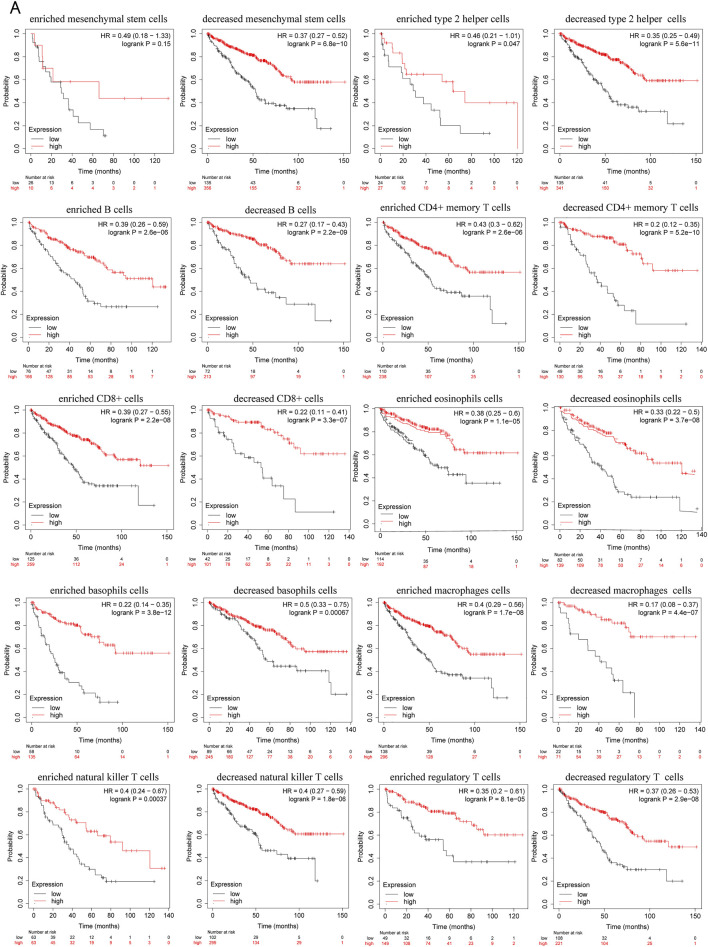
Kaplan–Meier survival plots comparing the low and high expression of PRKCE in different types of immune cell subgroups in the Kaplan–Meier plotter. **(A)** overall survival curves display the prognostic value of PRKCE expression based on different immune cell subgroups in Kaplan-Meier Plotter.

Since decreased expression of PRKCE is significantly correlated with immune infiltration and poor prognosis in KIRC. Thus, we propose a hypothesis that PRKCE may affect the prognosis of KIRC patients partly through immune infiltration. We investigated whether PRKCE expression affects the prognosis of KIRC through immune infiltration. The Kaplan-Meier plotter prognosis analyses based on the expression levels of PRKCE in KIRC in related immune cell subgroups unraveled that decreased expression of PRKCE and enriched infiltration of Type 2 T helper cells (*p* = 0.047), B cells (*p* = 2.6e-06), CD4^+^ memory T cells (*p* = 2.6e-06), CD8^+^ T cells (*p* = 2.2e-08), eosinophils cells (*p* = 1.1e-05), basophils cells (*p* = 3.8e-12), macrophages (*p* = 1.7e-08), natural killer T cells (*p* = 0.00037), regulatory T cells (*p* = 8.1e-05) cohort had a worse prognosis (Figure 8A). Similarly, the low expression of PRKCE in KIRC had poor prognosis in decreased Type 2 T helper cells (*p* = 5.6e-11), mesenchymal stem cells (*p* = 6.8e-10), B cells (*p* = 2.2e-09), CD4^+^ memory T cells (*p* = 5.2e-10), CD8^+^ T cells (*p* = 3.3e-07), eosinophils cells (*p* = 3.7e-08), basophils cells (*p* = 0.00067), macrophages (*p* = 4.4e-07), natural killer T cells (*p* = 1.8e-06), regulatory T cells (*p* = 2.9e-08) (Figure 8A). However, there was no significant difference between the high and low PRKCE expression groups’ overall survival in enriched mesenchymal stem cells (*p* = 0.15) (Supplementary Figure S6). These results indicate that decreased expression of PRKCE may affect the prognosis of KIRC patients in part due to immune cell infiltration.

## Discussion

KIRC is a highly aggressive cancer subtype of renal cell carcinoma due to resistance to chemotherapies and radiotherapy, which may decrease the survival rate of patients with advanced KIRC. Exploring the molecular mechanism of KIRC development and finding effective early diagnostic and therapeutic biomarkers may benefit KIRC patients. PKCε is associated with the initiation and aggressive phenotype of KIRC (Huang et al., 2011). Protein kinase C epsilon was a new target to control inflammation and immune-mediated disorders (Engers et al., 2000). An in-depth study of the expression, prognosis, and mechanism of PRKCE decreased expression and the association of PRKCE with tumor immune infiltration in KIRC can further reveal the cause of immunotherapy unresponsiveness and provide clues for KIRC immunotherapy.

The previous study found PKC epsilon (ε) upregulated in KIRC and was associated with tumor grade and T stage in clear cell RCC (ccRCC), but the mechanism of PKC epsilon (ε) increased expression in ccRCC is not fully clarified, interestingly, the protein level of PRKCE was weakly expressed in 786-O, OS-RC-2, SN12C, and SKRC39 cell lines (Huang et al., 2011). In our analysis, for the first time, we found that PRKCE mRNA levels were significantly low in KIRC. Decreased PRKCE expression was associated with unfavorable prognosis and poor clinicopathological characteristics, including high T stage, high histologic grade, advanced AJCC stage, and distant metastasis. We demonstrated that PRKCE has a high ROC score with an AUC of 0.791 for KIRC in the TCGA database. Considering that PRKCE is an effective prognostic factor, we constructed a nomogram combining the clinical data and PRKCE expression. The nomogram results showed that the C-index of the model was 0.760 (0.742–0.778), which indicates that this is a moderately accurate prediction model. In addition, our model could be a novel approach to evaluating the prognosis of clinicians in the future.

To further clarify the function of PRKCE in KIRC, we performed data analysis using GeneMANIA and CancerSEA. CancerSEA analysis results indicated that PRKCE might promote KIRC development and progression by regulating proliferation, inflammation, invasion, and metastasis. Furthermore, the impact of PRKCE on the ability of proliferation and invasion of KIRC cells was explored *in vitro*. We found that the malignant phenotype of KIRC cells was suppressed when PRKCE was overexpressed, indicating the expression of PRKCE is a potent target for kidney renal clear cell carcinoma therapy. We also used the STRING database to analyze the protein-protein interaction network of PRKCE. The “Cluster Profiler” R package and the “ggplot2″ R package were performed for GO and KEGG pathway analyses. These findings showed that PRKCE is involved in the immune response-activating cell-surface receptor signaling pathway, innate immune response activating cell-surface receptor signaling pathway, activation of innate immune responses, and immune response-regulating cell-surface receptor signaling pathway involved in phagocytosis, chemokine signaling pathway, T cell receptor signaling pathway, and PD-L1 expression and PD-1 checkpoint pathway in cancer (Supplementary Table S4), which demonstrated that PRKCE plays a pivotal immune regulation role in KIRC.

Limited evidence has clarified the mechanism for PRKCE downregulation in kidney renal clear cell carcinoma. We further used multiple online databases to investigate the underlying mechanism of low PRKCE mRNA in KIRC. Through cBioPortal analysis, we found no correlation between PRKCE decreased expression and copy number alterations in KIRC. Epigenetic modifications, such as DNA methylation, play a vital role in regulating gene expression (Yan et al., 2020). Aberrant promoter hypermethylation, often correlated with gene silencing, is vital in developing KIRC (Morris and Maher, 2010). In the present study, we reported that the level of PRKCE promoter methylation was significantly higher, and the expression of PRKCE was significantly lower in KIRC than in normal tissues, indicating that the decreased expression of PRKCE was regulated by high DNA methylation in KIRC. In addition, we further analysis found that the expression of PRKCE was negatively correlated with methylation sites cg04702872, cg04756223, and cg12428440. Thus, our results demonstrated that PRKCE decreased expression might be partially related to PRKCE hypermethylation.

Given that ncRNAs, including miRNAs, lncRNAs, and circular RNAs (circRNAs), participated in regulating gene expression (Fabrizio et al., 2020; Ghafouri-Fard et al., 2020; Lou et al., 2020). To investigate whether upstream miRNA regulated PRKCE low expression. We employed the starBase database and miRactDB to predict upstream binding miRNA of PRKCE. After conducting correlation analysis, binding sites analysis, expression analysis, and survival analysis, hsa-miR-21-5p was identified as the possible upstream binding miRNA of PRKCE. Meanwhile, hsa-miR-21-5p showed higher expression in KIRC than in the normal group. These data indicate that high DNA methylation and increased hsa-miR-21-5p correlate with PRKCE decreased expression in KIRC.

Tumor-infiltrating immune cells are an important part of the tumor microenvironment, and they play vital roles in immunotherapy responsiveness and promoting cancer development in KIRC (Vuong et al., 2019). Tumor-infiltrating immune cells (TIICs) are vital determinants of prognosis factors in KIRC (Zhang et al., 2019). CD8 T cells, Tregs, macrophages M0, and M2 infiltration were significantly associated with the poor prognosis of KIRC patients. In addition, activated memory CD4 T cells, Tfh cells infiltrations consistent with tumor T&M and stage trend, and M1 macrophages were correlated with distant tumor metastasis (Tabei et al., 2019; Wang et al., 2021). It has been found that NK T cells, tumor-associated macrophages, and dendritic cells often infiltrated KIRC form a unique TME because of a high infiltration of CD8^+^ T cells, and activated dendritic cells were often associated with longer survival rates in several solid tumors except for KIRC (Borcherding et al., 2021). Infiltrating macrophages by activating the AKT/mTOR signal induces EMT, increasing KIRC (Yang et al., 2016). In this study, we investigated the correlation between decreased expression of PRKCE and tumor-infiltration immune cells. We found that the TME with low expression of PRKCE contained higher levels of Tregs, cytotoxic cells, CD8 T cells, activated dendritic cells, higher ImmuneScore, and EstimateScore in KIRC. However, the expression of PRKCE was positively related to T help cells, mast cells, neutrophils, and eosinophils in KIRC. Through TIMER2.0 analysis, we found that the expression of PRKCE was significantly negatively correlated with macrophage infiltration. In summary, these results together suggested that decreased expression of PRKCE impacts the changes in the modulation of immune infiltration cells, promoting invasion and metastasis and poor prognosis of KIRC.

It is well known that chemokines play a vital role in promoting metastasis and infiltration of immune cells (Marcuzzi et al., 2018). The KEGG analysis showed that PRKCE was involved in the chemokine signaling pathway (Supplementary Figure S3L). Thus, we speculated that PRKCE might mediate immune cell infiltration through chemokine regulation. Recent studies found that 4 chemokines, including CCL4, CCL5, CXCL13, and XCL2, are abnormally expressed and correlated with immune infiltration cells in KIRC (Tan P. et al., 2021). The latest study found that high expression of CXCL13 increased migration in KIRC cell lines (Jiao et al., 2020). In addition, CXCL13 is associated with CD8T^+^ cell infiltration and is identified as a potential immunotherapeutic target marker in KIRC (Dai et al., 2021). Our study consistently confirmed that hsa-miR-21-5p was positively related to CCL4, CCL5, CXCL13, and XCL2. On the contrary, the expression of PRKCE was negatively correlated with CCL5, CXCL13, and XCL2 (all *p* < 0.05). These findings suggest that the PRKCE/hsa-miR-21-5p axis influences the infiltration of immune cells via chemokines CCL5, CXCL13, and XCL2.

Despite the infiltration of tumor immune cells that could affect the efficacy of immunotherapy, the expression level of immune checkpoints also influenced the response of immunotherapy (Chae et al., 2018). Immune checkpoint blockade therapy has been applied in KIRC patients (Bi et al., 2021). We further assessed the relationship between PRKCE and immune checkpoints. Low expression of PRKCE was significantly correlated with immune checkpoints (PDCD1, CTLA4, LAG3, and KLRB1) in KIRC. These results further support that PRKCE expression is significantly associated with immune cell infiltration and demonstrate that PRKCE plays a vital role in immune escape in the KIRC microenvironment. More importantly. We also assessed the prognostic value of PRKCE expression according to different immune cell subgroups in KIRC patients. The results showed that PRKCE influences the survival rate of KIRC patients partially through the infiltration of immune cells (Figure 8).

**FIGURE 9:**
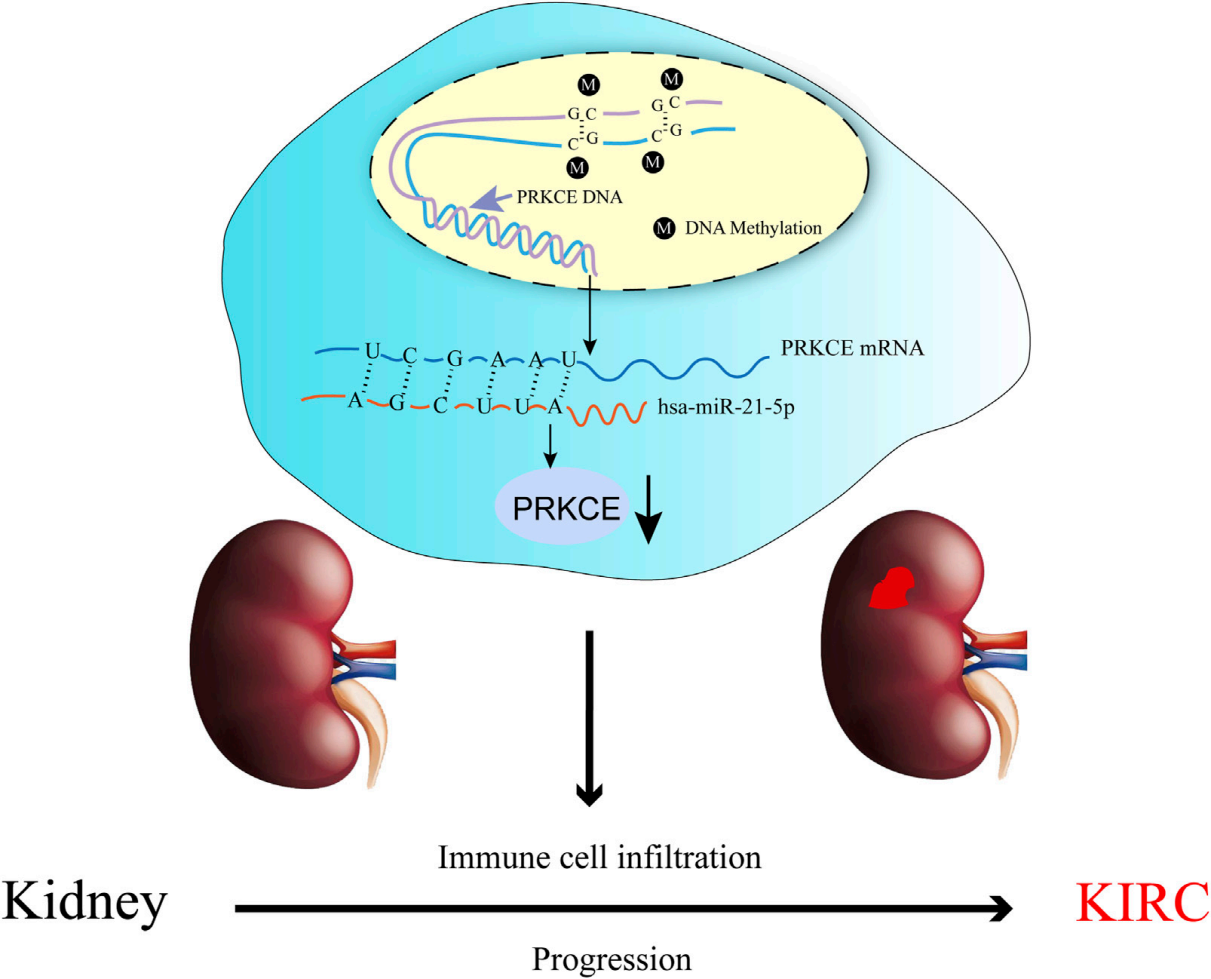
The model of hypermethylation and hsa-miR-21-5p–mediated downregulation of PRKCE in carcinogenesis of KIRC.

In conclusion, we reported that the decreased expression of PRKCE was significantly correlated with the progression, poor survival, and immune infiltration of KIRC, which might promote tumorigenesis through abnormal inflammation, methylation level, miRNA expression, and immune response. Furthermore, we also showed that a potential mechanism of PRKCE low expression in KIRC. The high methylation level of PRKCE and increased hsa-miR-21-5p in the regulation of PRKCE decreased expression in KIRC (Figure 9). We found overexpression of PRKCE significantly suppressed the proliferation, invasion, and migration of KIRC cells *in vitro*. However, more basic experimental studies and large clinical trials should be further validated. PRKCE may be used as a clinical diagnostic biomarker and an effective immunotherapy target.

## Data Availability

The datasets presented in this study can be found in online repositories. The names of the repository/repositories and accession number(s) can be found in the article/Supplementary Material.
