# Supplementary material for: miR-21-5p/PRKCE axis implicated in immune infiltration and poor prognosis of kidney renal clear cell carcinoma
Source: Front Genet. 2022 Sep 13;13:978840. doi: 10.3389/fgene.2022.978840 (PMC9516396; doi:10.3389/fgene.2022.978840)
Supplement: Supplementary file 1 [file DataSheet1.PDF]

Supplementary Figure 1

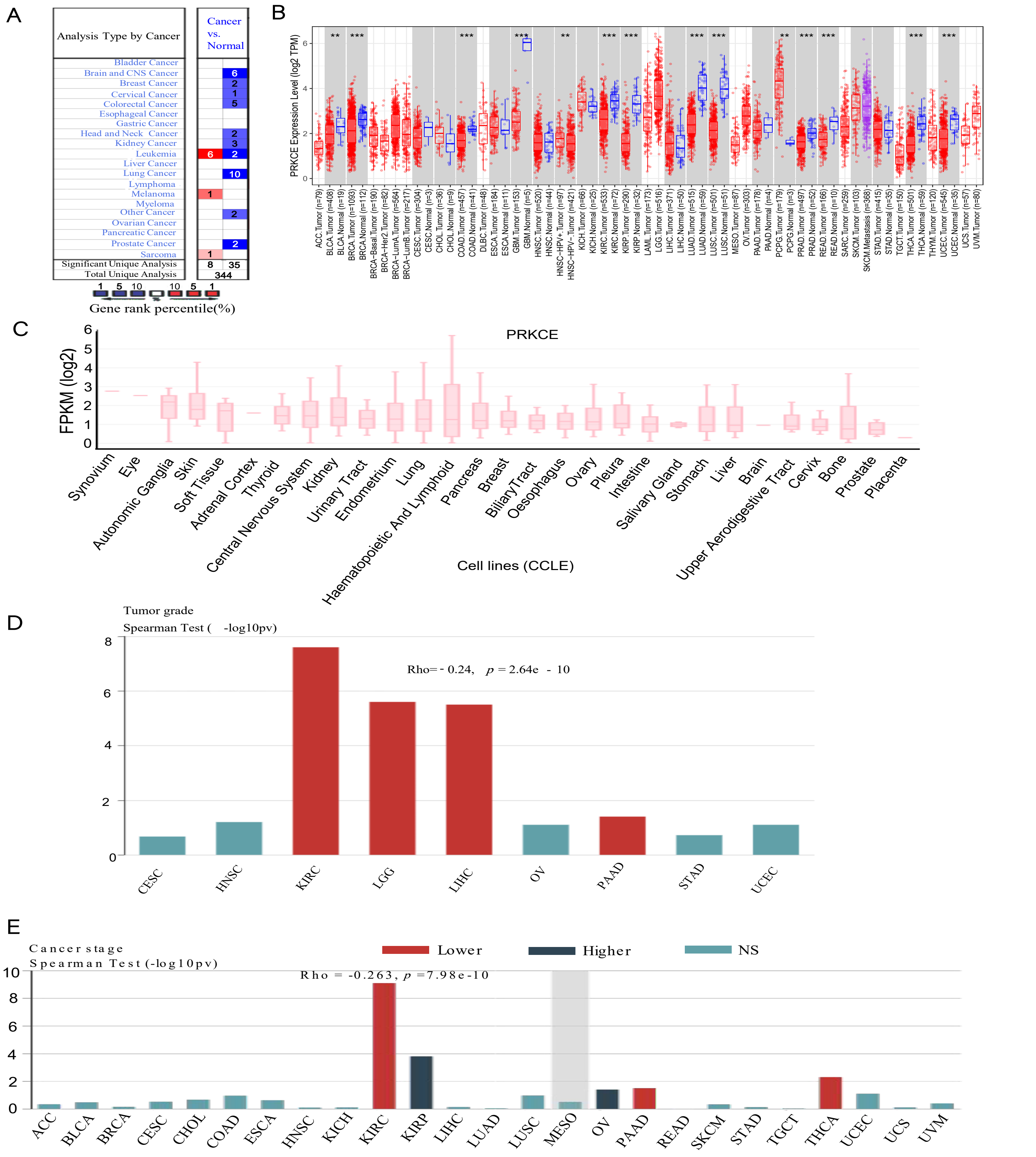


**Supplementary Figure 1**. The Expression levels of PRKCE in various cancers

(**A**) The expression of PRKCE in various cancer tissues compared with normal tissues in Oncomine database.

(**B**) The expression of PRKCE in pan-cancer investigated by TIMER2.0 dataset (***p <0.001, ** p < 0.01, *p < 0.05,).

(**C**) The expression of PRKCE in pan-cancer cell line based on CCLE. (**D**) Associations between PRKCE expression and tumor grade across human cancers in TISIDB. (**E**) Associations between PRKCE expression and cancer stage across human cancers in TISIDB.

Supplementary Figure 2

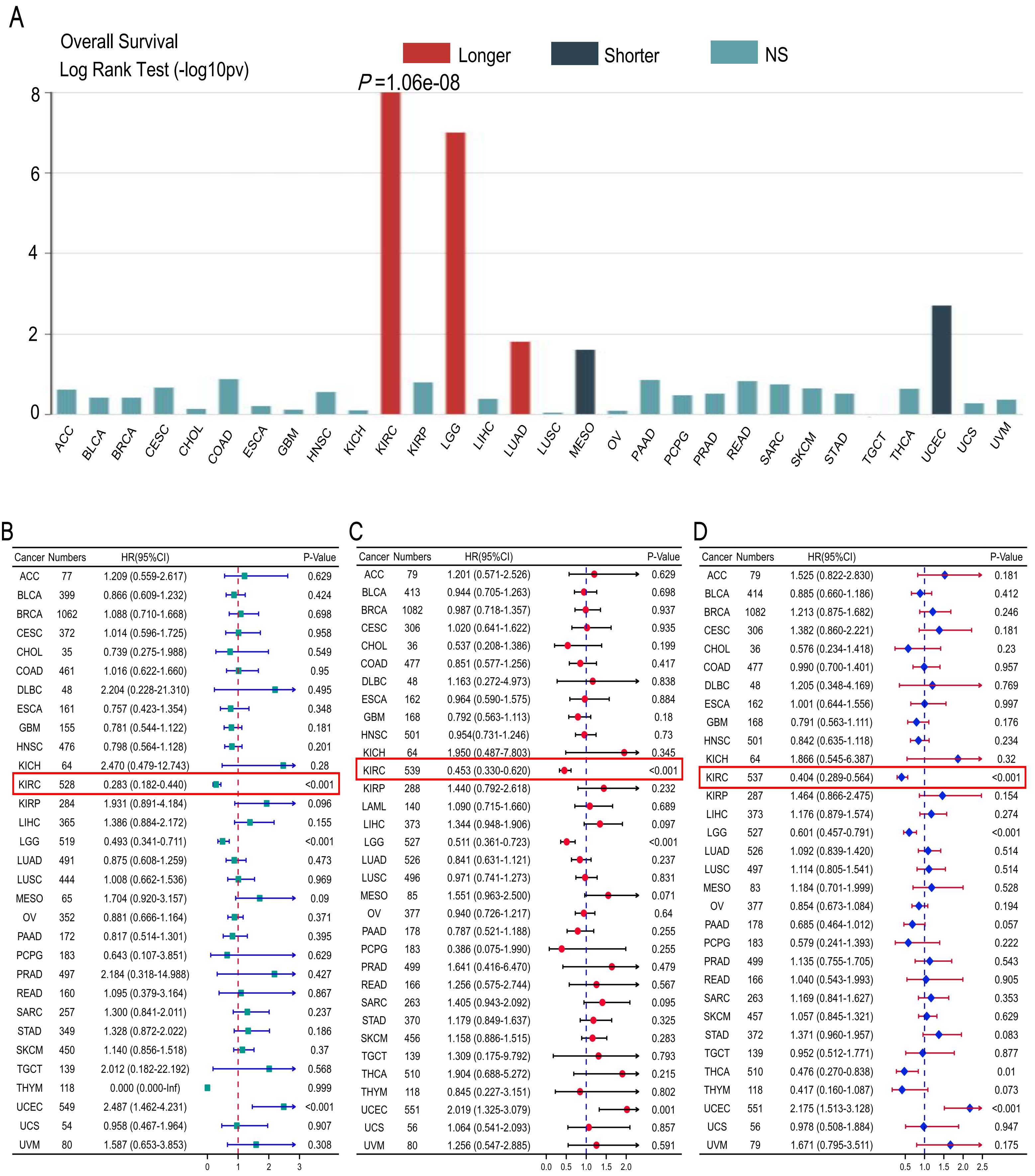


**Supplementary Figure 2.** The effect of PRKCE on the prognosis of various cancers using Cox proportional hazards models and it's clinical features in TISIDB and Forest plots.(**A**) Overall survival analysis comparing the high and low expression of PRKCE in different types of cancer in TISIDB. (**B-D**) Forest plots displaying relation between PRKCE expression and patient prognosis (OS, DSS and PFI) of different cancers in TCGA database.

Supplementary Figure 3

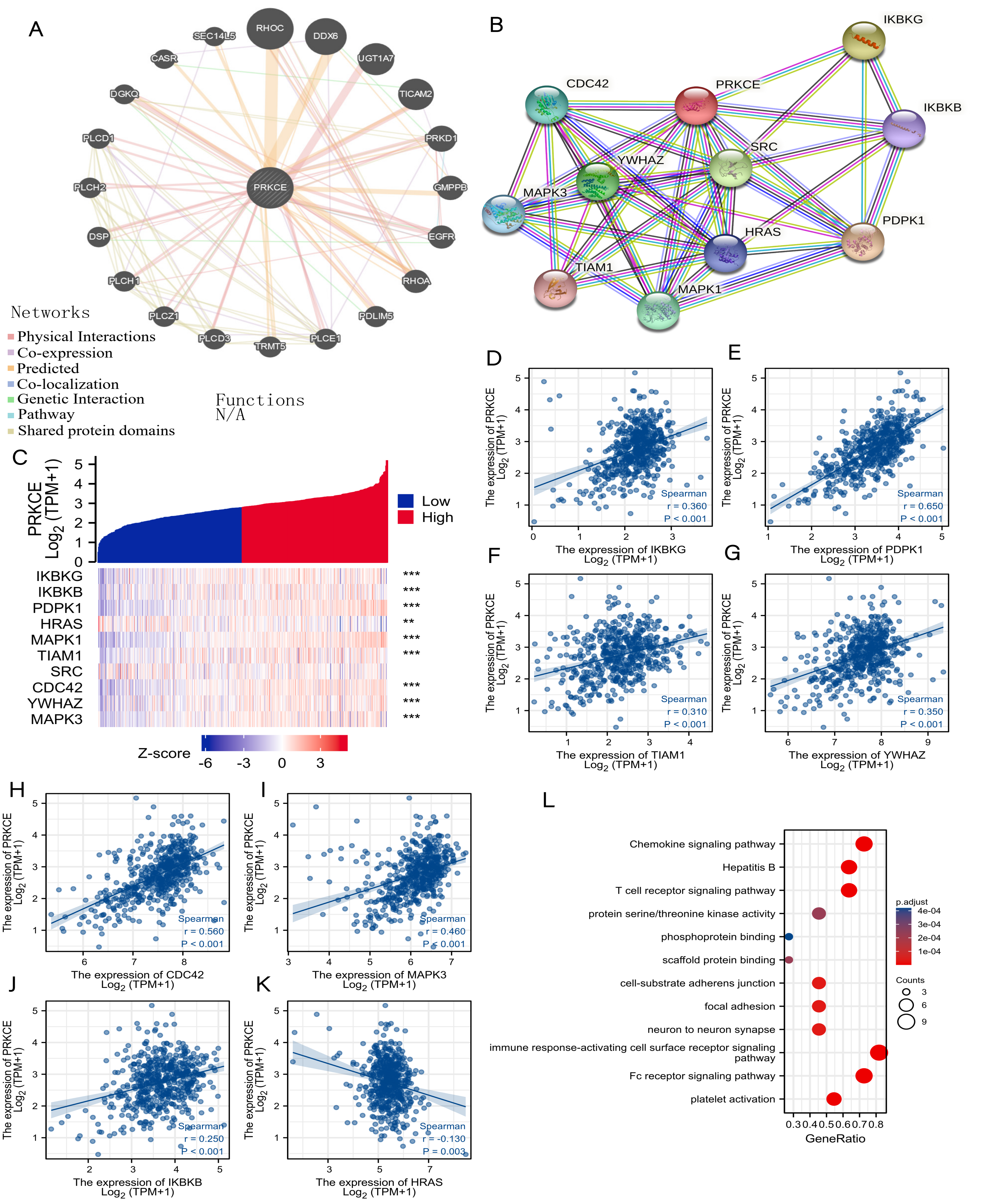


**Supplementary Figure 3**. PPI networks and functional enrichment analyses

(**A**)The gene-gene interaction network of PRKCE was generated using GeneMANIA. (**B**) The PPI network of PRKCE was shown based on STRING. (**C**) A heat map displays co-expressed genes in PRKCE-low and –high groups. (**D-K**) Relations between the expression of PRKCE and the co-expressed genes in KIRC. (**L**) GO and KEGG enrichment analyses of 10 involved genes. PRKCE was associated with the Chemokine signaling pathway, immune response-activating cell-surface receptor signaling pathway, and T cell receptor signaling pathway.

Supplementary Figure 4

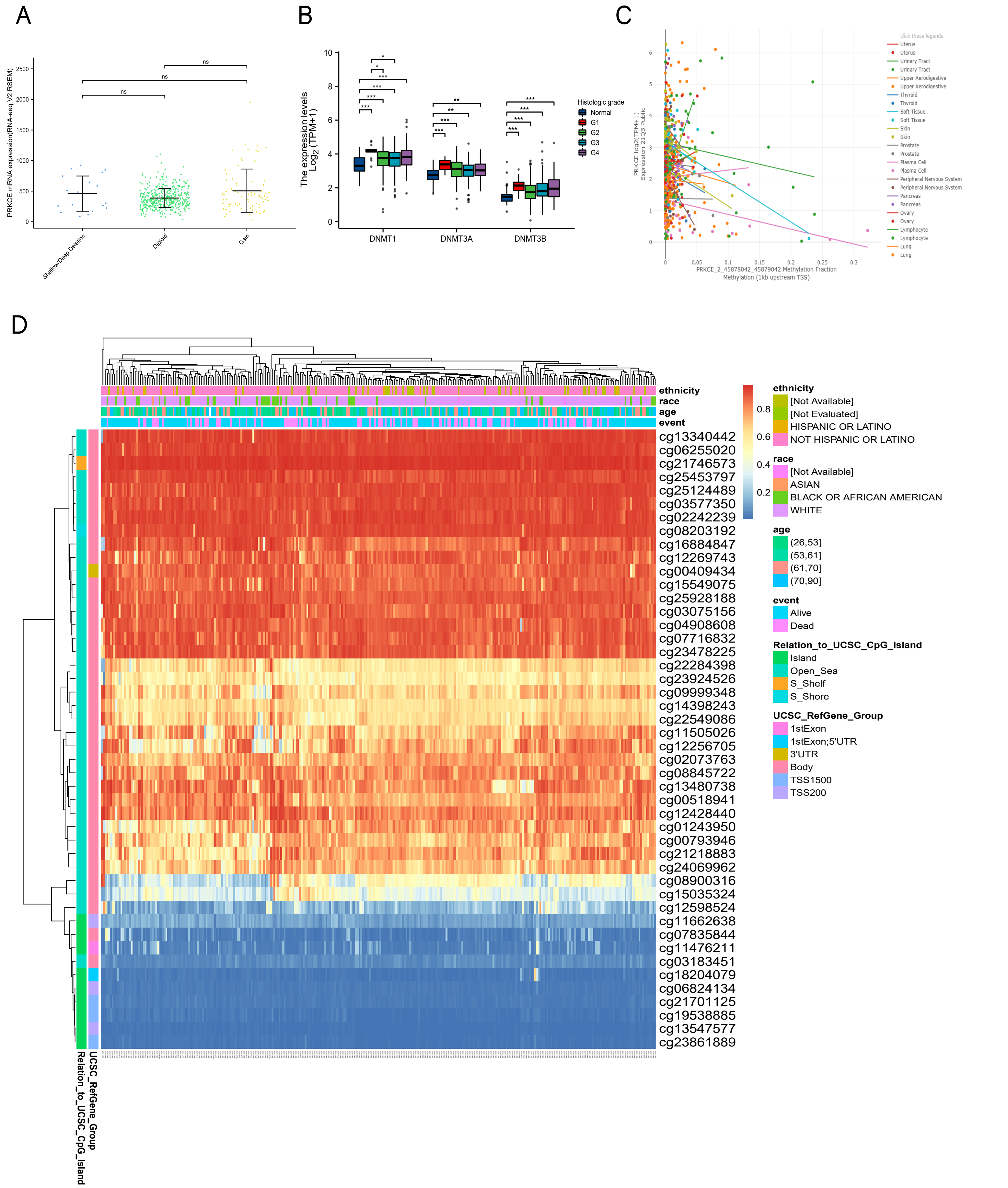


**Supplementary Figure 4.** Methylation analysis of PRKCE in KIRC
(**A**) The expression level in different CNV of PRKCE. (**B**) DNMT1, DNMT3A, DNMT3B expression in different histologic grades of KIRC. (**C**) The correlation between PRKCE methylation and its expression level analysis by using CCLE data in the various cancer cell. (**D**) The visualization between the PRKCE expression and the methylation level.

Supplementary Figure 5

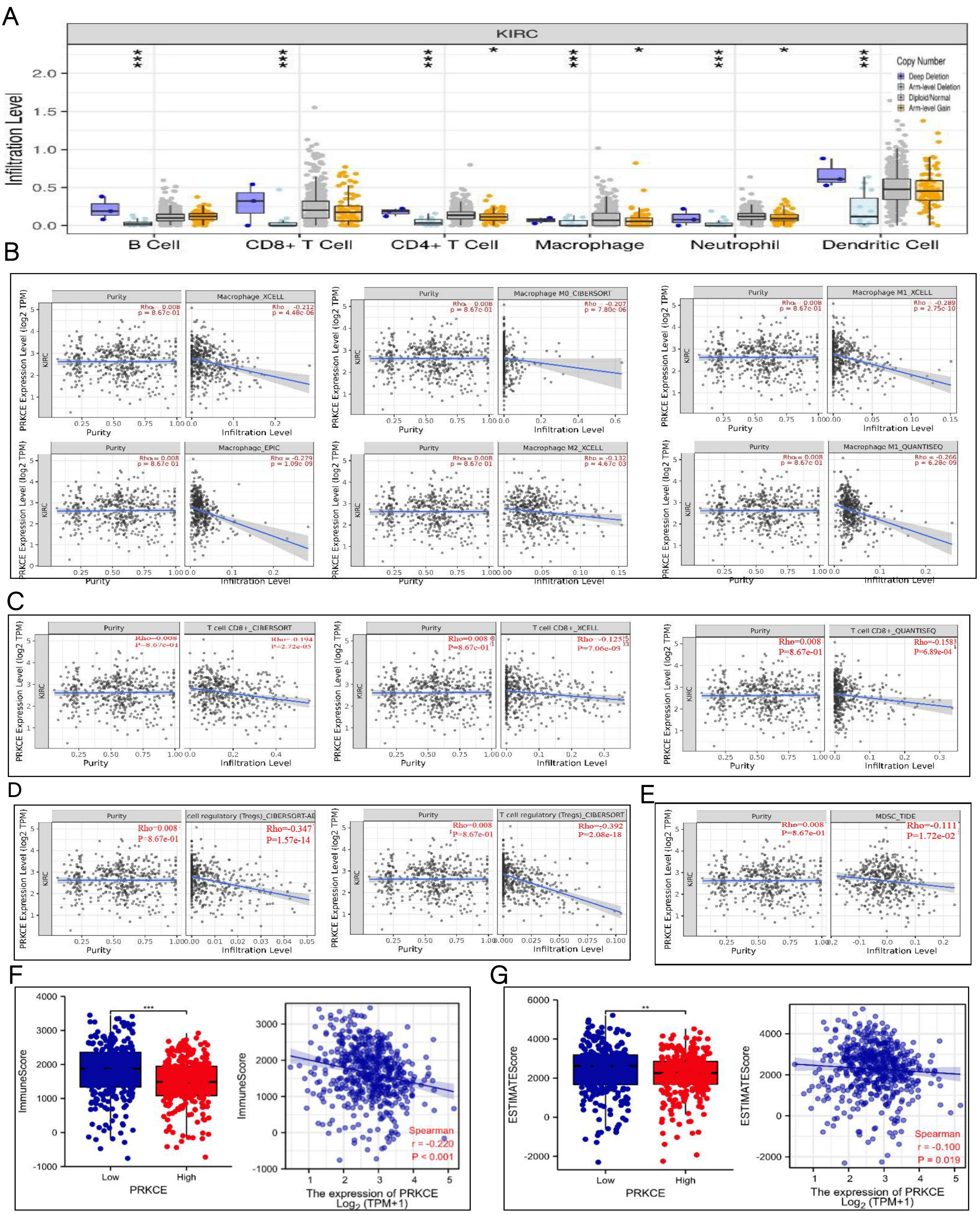


**Supplementary Figure 5**.The relationship of immune cell infiltration with PRKCE level in KIRC. (**A**) The infiltration level of various immune cells under different copy numbers of PRKCE in KIRC. (**B-D**) PRKCE expression significantly negatively correlated with the infiltration of (**B**) macrophage, (**C**) CD8+ T cell, and (D) Tregs, but not with tumor purity. (**E**) The relationship between MDSC and infiltration levels. (**F,G**) The expression of PRKCE was negatively correlated with ImmuneScore and EsimateScore.

Supplementary Figure 6

| Subgroup | Numbers | HR(95%CI) | P−Value |
|---|---|---|---|
| B cells | | | |
| enriched | 242 | 0.39(0.26−0.59) | 2.6e−06 |
| decreased | 285 | 0.27(0.17−0.43) | 2.2e−09 |
| CD4+memory T−cells | | | |
| enriched | 348 | 0.43(0.3−0.62) | 2.6e−06 |
| decreased | 179 | 0.2(0.12−0.35) | 5.2e−10 |
| Eosinophils cells | | | |
| enriched | 306 | 0.38(0.25−0.6) | 1.1e−05 |
| decreased | 221 | 0.33(0.22−0.5) | 3.7e−08 |
| CD8+ memory T−cells | | | |
| enriched | 384 | 0.39(0.27−0.55) | 2.2e−08 |
| decreased | 143 | 0.22(0.11−0.41) | 3.3e−07 |
| Basophils cellS | | | |
| enriched | 193 | 0.22(0.14−0.35) | 3.8e−12 |
| decreased | 334 | 0.5(0.33−0.75) | 0.00067 |
| Macrophages | | | |
| enriched | 434 | 0.4(0.29−0.56) | 1.7e−08 |
| decreased | 93 | 0.17(0.08−0.37) | 4.4e−07 |
| Regulatory T−cells | | | |
| enriched | 198 | 0.35(0.2−0.61) | 8.1e−05 |
| decreased | 329 | 0.37(0.26−0.53) | 2.9e−08 |
| Natural killer T−cells | | | |
| enriched | 126 | 0.4(0.24−0.67) | 0.00037 |
| decreased | 401 | 0.4(0.27−0.59) | 1.8e−06 |
| Type 2 T−helper cells | | | |
| enriched | 51 | 0.46(0.21−1.01) | 0.047 |
| decreased | 476 | 0.35(0.25−0.49) | 5.6e−11 |
| Masenchymal stem cells | | | |
| enriched | 36 | 0.49(0.18−1.33) | 0.15 |
| decreased | 491 | 0.37(0.27−0.52) | 6.8e−10 |

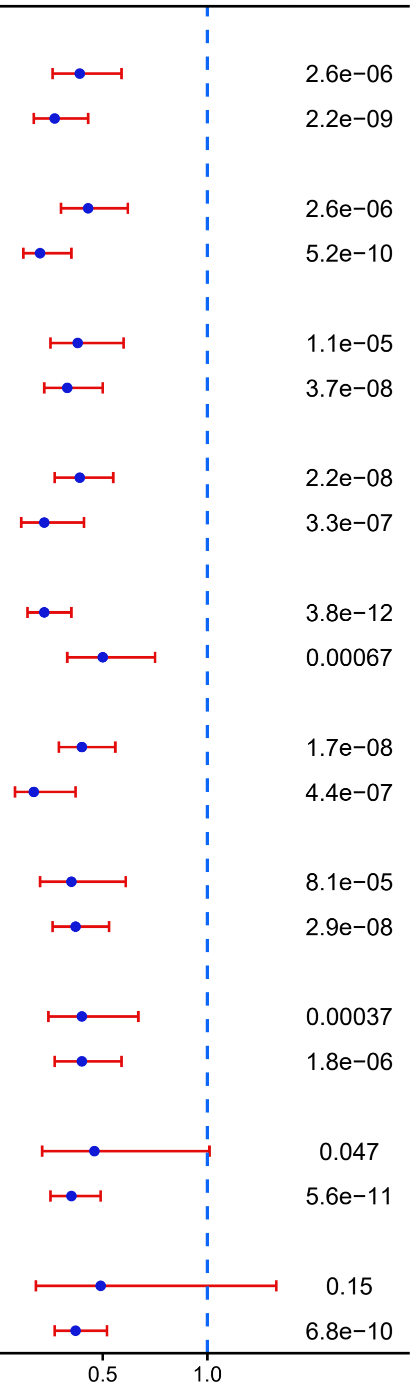


**Supplementary Figure 6**. The forest plot of PRKCE expression and immune cell subgroups in KIRC Correlations between PRKCE expression and OS in different immune cell subgroups in KIRC patients were investigated by Kaplan-Meier plotter.

**Table S1. The correlation between clinicopathological variables and PRKCE expression**

| Characteristics | levels | Low expression of PRKCE | High expression of PRKCE | p |
|---|---|---|---|---|
| n | | 269 | 270 | |
| T stage, n (%) | T1 | 112 (20.8%) | 166 (30.8%) | < 0.001 |
| | T2 | 40 (7.4%) | 31 (5.8%) | |
| | T3 | 111 (20.6%) | 68 (12.6%) | |
| | T4 | 6 (1.1%) | 5 (0.9%) | |
| N stage, n (%) | N0 | 121 (47.1%) | 120 (46.7%) | 0.238 |
| | N1 | 11 (4.3%) | 5 (1.9%) | |
| M stage, n (%) | M0 | 196 (38.7%) | 232 (45.8%) | < 0.001 |
| | M1 | 54 (10.7%) | 24 (4.7%) | |
| Pathologic stage, n (%) | Stage I | 109 (20.3%) | 163 (30.4%) | < 0.001 |
| | Stage II | 30 (5.6%) | 29 (5.4%) | |
| | Stage III | 72 (13.4%) | 51 (9.5%) | |
| | Stage IV | 56 (10.4%) | 26 (4.9%) | |
| Primary therapy outcome, n (%) | PD | 7 (4.8%) | 4 (2.7%) | 0.185 |
| | SD | 1 (0.7%) | 5 (3.4%) | |
| | PR | 0 (0%) | 2 (1.4%) | |
| | CR | 54 (36.7%) | 74 (50.3%) | |

| Characteristics | levels | Low expression of PRKCE | High expression of PRKCE | p |
|---|---|---|---|---|
| Gender, n (%) | Female | 68 (12.6%) | 118 (21.9%) | < 0.001 |
| | Male | 201 (37.3%) | 152 (28.2%) | |
| Race, n (%) | Asian | 4 (0.8%) | 4 (0.8%) | 0.570 |
| | Black or African American | 32 (6%) | 25 (4.7%) | |
| | White | 229 (43%) | 238 (44.7%) | |
| Age, mean ± SD | | 61.13 ± 11.99 | 60.13 ± 12.19 | 0.339 |

**Table S2. PRKCE expression association with clinical-pathological characteristics (logistic regression)**

| Characteristics | Total(N) | Odds Ratio(OR) | P value |
|---|---|---|---|
| T stage (T3 vs. T1) | 457 | 0.413 (0.280-0.606) | <0.001 |
| N stage (N1 vs. N0) | 257 | 0.458 (0.141-1.301) | 0.159 |
| M stage (M1 vs. M0) | 506 | 0.375 (0.221-0.623) | <0.001 |
| Pathologic stage (Stage II&Stage III vs. Stage I) | 454 | 0.524 (0.358-0.766) | <0.001 |
| Histologic grade (G4 vs. G1) | 89 | 0.261 (0.073-0.840) | 0.028 |
| Gender (Male vs. Female) | 539 | 0.436 (0.301-0.626) | <0.001 |
| Race (White vs. Asian&Black or African American) | 532 | 1.290 (0.767-2.186) | 0.338 |
| Age (>60 vs. <=60) | 539 | 0.831 (0.592-1.164) | 0.282 |

**Table S3. Univariate and multivariate survival methods (Overall Survival) of prognostic covariates in patients with KIRC**

| Characteristics | Total(N) | Univariate analysis | | Multivariate analysis | |
|---|---|---|---|---|---|
| | | Hazard ratio (95% CI) | P value | Hazard ratio (95% CI) | P value |
| T stage | 539 | | | | |
| T1&T2 | 349 | Reference | | | |
| T3&T4 | 190 | 3.228 (2.382-4.374) | **<0.001** | 1.580 (0.693-3.603) | 0.276 |
| N stage | 257 | | | | |
| N0 | 241 | Reference | | | |
| N1 | 16 | 3.453 (1.832-6.508) | **<0.001** | 1.561 (0.776-3.142) | 0.212 |
| M stage | 506 | | | | |
| M0 | 428 | Reference | | | |
| M1 | 78 | 4.389 (3.212-5.999) | **<0.001** | 2.711 (1.604-4.583) | **<0.001** |
| Pathologic stage | 536 | | | | |
| Stage I&Stage II | 331 | Reference | | | |
| Stage III&Stage IV | 205 | 3.946 (2.872-5.423) | **<0.001** | 1.222 (0.484-3.085) | 0.671 |
| Histologic grade | 531 | | | | |
| G1&G2 | 249 | Reference | | | |
| G4&G3 | 282 | 2.702 (1.918-3.807) | **<0.001** | 1.477 (0.883-2.471) | 0.137 |
| Age | 539 | | | | |
| <=60 | 269 | Reference | | | |
| >60 | 270 | 1.765 (1.298-2.398) | **<0.001** | 1.599 (1.042-2.453) | **0.032** |

| Characteristics | Total(N) | Univariate analysis | | Multivariate analysis | |
|---|---|---|---|---|---|
| | | Hazard ratio (95% CI) | P value | Hazard ratio (95% CI) | P value |
| PRKCE | 539 | | | | |
| Low | 270 | Reference | | | |
| High | 269 | 0.453 (0.330-0.620) | **<0.001** | 0.571 (0.357-0.914) | **0.020** |

## Table S4. Gene ontology (GO) enrichment and Kyoto Encylopedia of Genes and Genomes (KEGG) pathway analyses of co-expression genes

| ONTOLOGY | ID | Description | GeneRatio | BgRatio | pvalue | p.adjust | qvalue |
|---|---|---|---|---|---|---|---|
| BP | GO:0038093 | Fc receptor signaling pathway | 8/11 | 241/18670 | 1.10e-13 | 1.24e-10 | 3.73e-11 |
| BP | GO:0002429 | immune response-activating cell surface receptor signaling pathway | 9/11 | 473/18670 | 2.10e-13 | 1.24e-10 | 3.73e-11 |
| BP | GO:0030168 | platelet activation | 6/11 | 153/18670 | 1.23e-10 | 3.99e-08 | 1.20e-08 |
| BP | GO:0007596 | blood coagulation | 7/11 | 336/18670 | 1.78e-10 | 3.99e-08 | 1.20e-08 |
| BP | GO:0007599 | hemostasis | 7/11 | 341/18670 | 1.98e-10 | 3.99e-08 | 1.20e-08 |
| BP | GO:0002220 | innate immune response activating cell surface receptor signaling pathway | 5/11 | 116/18670 | 3.81e-09 | 5.02e-07 | 1.50e-07 |
| BP | GO:0002433 | immune response-regulating cell surface receptor signaling pathway involved in phagocytosis | 5/11 | 139/18670 | 9.48e-09 | 6.96e-07 | 2.09e-07 |
| BP | GO:0002218 | activation of innate immune response | 6/11 | 319/18670 | 1.02E-08 | 6.96E-07 | 2.09E-07 |
| CC | GO:0098984 | neuron to neuron synapse | 5/11 | 350/19717 | 7.25e-07 | 3.40e-05 | 1.41e-05 |
| CC | GO:0005925 | focal adhesion | 5/11 | 405/19717 | 1.49e-06 | 3.40e-05 | 1.41e-05 |
| CC | GO:0005924 | cell-substrate adherens junction | 5/11 | 408/19717 | 1.54e-06 | 3.40e-05 | 1.41e-05 |
| CC | GO:0030055 | cell-substrate junction | 5/11 | 412/19717 | 1.62e-06 | 3.40e-05 | 1.41e-05 |

| ONTOLOGY | ID | Description | GeneRatio | BgRatio | pvalue | p.adjust | qvalue |
|---|---|---|---|---|---|---|---|
| CC | GO:0005901 | caveola | 3/11 | 80/19717 | 1.04e-05 | 1.73e-04 | 7.16e-05 |
| MF | GO:0004674 | protein serine/threonine kinase activity | 5/11 | 439/17697 | 3.75e-06 | 2.25e-04 | 7.80e-05 |
| MF | GO:0097110 | scaffold protein binding | 3/11 | 59/17697 | 5.70e-06 | 2.25e-04 | 7.80e-05 |
| MF | GO:0051219 | phosphoprotein binding | 3/11 | 83/17697 | 1.60e-05 | 4.21e-04 | 1.46e-04 |
| MF | GO:0004707 | MAP kinase activity | 2/11 | 14/17697 | 3.18e-05 | 6.29e-04 | 2.18e-04 |
| MF | GO:0004708 | MAP kinase kinase activity | 2/11 | 16/17697 | 4.20e-05 | 6.63e-04 | 2.30e-04 |
| KEGG | hsa04062 | Chemokine signaling pathway | 8/11 | 192/8076 | 1.37e-11 | 1.07e-09 | 1.04e-10 |
| KEGG | hsa04660 | T cell receptor signaling pathway | 7/11 | 104/8076 | 1.52e-11 | 1.07e-09 | 1.04e-10 |
| KEGG | hsa05161 | Hepatitis B | 7/11 | 162/8076 | 3.54e-10 | 1.67e-08 | 1.62e-09 |
| KEGG | hsa05215 | Prostate cancer | 6/11 | 97/8076 | 1.13e-09 | 3.74e-08 | 3.63e-09 |
| KEGG | hsa04625 | C-type lectin receptor signaling pathway | 6/11 | 104/8076 | 1.73e-09 | 3.74e-08 | 3.63e-09 |
| KEGG | hsa05235 | PD-L1 expression and PD-1 checkpoint pathway in cancer | 5/11 | 89/8076 | 6.37e-08 | 4.72e-07 | 4.58e-08 |
